# Development of Ionic Polymer-Functionalized Single Nanopipette Probes

**DOI:** 10.1021/acs.analchem.6c03481

**Published:** 2026-08-29

**Authors:** Kan Tang, Surendra R. Puri, Milton Ahamed, Ruoyu Yang, Dipak Baram, Ji-young Ock, Xi Chelsea Chen, Gangli Wang

**Affiliations:** † Department of Chemistry, 1373 Georgia State University, Atlanta, Georgia 30302, United States; # Chemical Sciences Division, Oak Ridge National Laboratory, Oak Ridge, Tennessee 37830, United States

## Abstract

Nanoprobes govern the spatiotemporal resolution of scanning probe microscopy techniques and are almost exclusively constructed with inorganic materials. Herein, we report the functionalization of single glass/quartz nanopipettes with lithium conducting polymers as conductive polymer nanoprobes. Lithium ion electrolytes with a reactive anion that can be covalently bonded (LiMTFSI) and a nonreactive anion (LiTFSI) are polymerized inside individual nanopipettes to develop single-ion conducting (p-SIC) and dual-ion conducting (p-DIC) nanoprobes, respectively. Customized tools and procedures are developed for handling delicate nanopipettes and for real-time monitoring of the polymerization reactions in an inert environment inside a glovebox. In situ monitoring of ionic conductivity changes characterizes the kinetics of polymerization in a single or bundle of nanopipettes. The room-temperature ionic conductivity and thermal activation energy of polymers inside nanopipettes are consistent with bulk values statistically, 10^–5^ S/cm and 36 ± 7 kJ/mol for p-SIC and 10^–4^ S/cm and 20 ± 4 kJ/mol for p-DIC, respectively. Heterogeneity among different polymer@nanopipettes is mitigated through an internal standardization method. Proof of principles for probing localized conductance, e.g., at the nanotip–substrate interface, are demonstrated using p-DIC@nanopipettes in repetitive approaching–landing–retrieval from a substrate immersed in lithium electrolyte solution. A common technical challenge with a risk of breaking the fragile nanotips is reduced by adopting a tilted nanotip—substrate alignment. The design also allows for closer proximity or surface contact theoretically limited by the quartz wall, which is critical for fundamental interfacial transport studies and for better imaging resolution. A capacitance term is found to be necessary in addition to the widely adopted tip—substrate distance-dependent resistance to quantitatively fit the approach curves. The polymer@nanotip platform provides a new capability to investigate ion transport across solid–electrolyte interfaces, which is vital for applications such as next-generation solid-state batteries, miniaturized memristors and transistors, and other energy and imaging systems.

## Graphical abstract

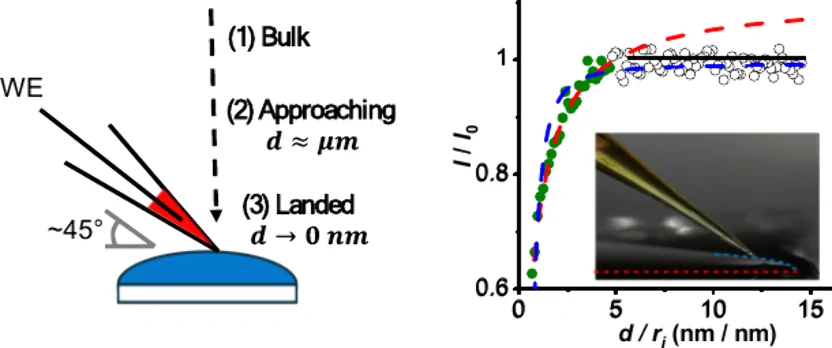

## Introduction

Ion transport (IT) at nanoscale interfaces is often the limiting factor in functionality and underpins the advances in batteries, electrochemical capacitors, and other energy conversion systems, ion separation, the development of miniaturized devices such as field effect transistors and ionic memristors, and electrochemical imaging and sensing technologies.^1–10^ Insights with greater spatiotemporal resolution, on IT at a particular interface deconvoluted from the bulk and other interfaces, are critical because (1) knowledge of the slowest rate-limiting step among sequential processes governing the overall conductivity may shine light on new mechanisms or strategies for improving functionality and (2) heterogeneity in materials, especially within solid-electrolyte interphase (SEI) structures, is fundamentally implicit in the aforementioned applications in terms of efficiency and capacity but inaccessible in ensemble measurements. Scanning ion conductance microscopy (SICM), among other scanning probe techniques, is uniquely suitable to studying charge transport at electrochemical interfaces.^11–13^ Prior work, however, is mostly restricted to aqueous or other solvated electrolyte systems in noncontact mode for nonintrusive imaging, while new functional nanoprobes for studying or adopting solid-state electrolytes (SSEs) remain to be developed.^14–16^

Polymer SSEs offer exciting promises over conventional solvated electrolytes with merits such as a larger electrochemical potential window and thus higher energy density, improved chemical stability for being less prone to leakage and fire,^17–20^ and mechanical flexibility circumventing the drawbacks of brittleness and poor interfacial contacts with electrodes in inorganic solid electrolytes.^21–23^ While polymer SSEs have relatively low ion conductivity historically,^3,24–26^ improvements of up to 10^–4^ to 10^–3^ S/cm (25 °C) have been achieved in several different groups of polymer matrixes, such as ethylene oxide-, ethylene glycol-, and methyl ether acrylate-derived PEO/PEGMEA,^27,28^ poly­(vinyl ethylene carbonate) (p-VEC),^19,29^ and poly-1,3-dioxolane (p-DOL).^30–32^ Typically, a dual ion conductor (DIC) such as lithium bis­(trifluoro­methane­sulfonyl)­imide (LiTFSI) salt is added to the polymer matrix in which both cations and anions are mobile charge carriers. For single ion conductors (SIC), for example, lithium sulfonyl-(trifluoromethane sulfonyl)­imide methacrylate (LiMTFSI), the anion can be copolymerized with VEC to form a polymerized anionic framework,^19^ gaining a higher cation transference number often at a compromise of lower ionic conductivity of 10^–5^ S/cm (25 °C).^33,34^

An essential property of SSEs is ionic conductivity, which can be measured with two electrodes between which a film of SSEs is sandwiched in. In view of a simplified equivalent circuit, the bulk SSE is in series with two electrode–SSE interfaces. The overall conductivity is analyzed with frequency-domain methods such as electrochemical impedance spectroscopy (EIS) or broadband dielectric spectroscopy (BDS), where an alternating current (AC) signal is invoked by an AC potential with small amplitudes and time-domain methods such as cyclic voltammetry (CV), where electron transfer (ET) is often involved but not required for DC conductivity.^19,21,35,36^ Polymer electrolytes have been incorporated into nanopipettes (NPs) for conductance-based biosensing and imaging applications, almost exclusively focusing on the external point of interest rather than the fundamental properties of polymer electrolytes themselves.^14–16^ The operation principle is that the ionic or redox current is limited by the nanotip, and the changes in the current signal encode spatiotemporal information about the point of interest, e.g., approaching a surface, binding dissociation, the translocation of matter, or biological cell activities.^37–40^

Rigorously controlled polymer derivatization inside single NPs offers much needed capability for fundamental studies of IT at the interface between polymer SSE and another phase of interest and for the effects of nanoconfinement in light of insights that the reaction kinetics and mechanism in microdroplets may deviate from those of bulk-phase reactions.^41,42^ Leveraging on our expertise on the IT dynamics at nanoscale interfaces, motivated by recent advances in polymer SSEs, we report herein the successful thermally activated radical polymerization of SIC and DIC inside individual conical nanopipettes, abbreviated as p-SIC@NP and p-DIC@NP hereafter. Complete polymerization throughout the conical nanotip is sharply distinct from previously adopted surface modification, cross-linking, or other assembly strategies where the transport-limiting nanotip is partially filled or occluded with polymer and solution IT remains predominant. Our design ensures that the IT at the SSE will be the signal-limiting step, not the monomer salt solution in unoccupied volume in the nanopipette or other sequential processes in the whole measurement circuits. This is because the applied potential is dropped primarily at the conical nanotip, establishing a strong localized electric field. A related advantage of the conical nanotip being the transport/signal-limiting step is that the blocking effects at the (macro)­electrode contact become negligible within the time and current scale of the measurements. The universal challenge of heterogeneity in nanodevice nanomaterials is mitigated by using the ionic conductivity of Li salt prior to polymerization as an internal standard. The efficacy of the polymerization confined in single nanopipettes is characterized with analytical tools and further validated by the consistency in the ionic conductivity and thermal activation energy barrier compared to those of bulk-synthesized samples. To showcase the application of polymer@NPs as scanning probes, reproducible approach curves were obtained through a nonvertical configuration that drastically reduced the risk of tip breakage. The distance between the nanotip and surface approached the fundamental limit of insulating glass wall thickness, about 20–50 nm depending on the nanotips used. In other words, greater resolution can be achieved in principle for advancing the aforementioned applications.

## Experimental Section

### Materials and Measurements

Details about the chemicals and materials, device fabrication, and measurements are provided in the Supporting Information (SI). Briefly, nanopipettes and micropipettes were fabricated from quartz capillaries using a P-2000 laser puller (Sutter Instrument Company). The pipettes were backfilled to load or replace solutions using an in-house-made microinjector and then centrifuged at 4500 rpm for about 2 min to fill the nanotip region as previously reported.^8^ Effective filling to the nanotip is confirmed by conductivity measurements, for example, after loading and immersion in 1 M KCl aqueous solution. The pipet size is characterized by conductivity measurements using eq 1, a widely adopted approach to address the uncertainties from fabrication and to mitigate the sacrificial nature of electron microscopy imaging^43–45^

$$R_{p}=\frac{1}{\sigma \left(KCl\right)r_{i}}\left(\frac{1}{\pi \tan\theta }+\frac{1}{4}\right)$$ 1

where *R*_*p*_ is the ohmic resistance (Ω) of the nanopore which can be determined from the linear current–potential (*I–V*) curves from CV measurements; σ is the ionic conductivity (S/cm) of the 1 M KCl solution (or other standard materials), σ­(KCl, 1 M) = 1.1 × 10^–1^ S/cm at 25 °C; *r*_*i*_ is the inner radius of a nanopipette (nm); and θ is the half cone angle (deg). The half cone angle of the conical pipettes varies from the end of the nanotip toward the stem and depends on pulling parameters but is in the general range of 8–11° in our fabrication determined by scanning electron microscopy (SEM).^8,46^

### Polymerization in Single NPs and In Situ Monitoring

Individual pipettes are generally characterized in 1 M KCl; they are washed thoroughly with DI water, with the effective removal of KCl salt confirmed by a decrease in current from CV,^8,46^ and dried in an oven before being transferred to a nitrogen-filled glovebox to load the reagents for polymerization. The ratio of the measured current or conductance from the same pipettes, with Li salt monomers versus 1 M KCl, eliminates the geometric factors in eq 1 and thus the heterogeneity among individual NPs. In other words, the conductivity of the Li salt monomer is first standardized by 1 M KCl by the current or conductance ratio for each pipet. The current changes associated with polymerization are monitored in situ in each synthesis, so normalization by the initial current prior to heating provides conductivity changes (as the remaining parameters remain unchanged). Each synthesis can include either one NP or a bundle of NPs, all protected with a plastic shroud to avoid tip damage during centrifugation and operation. The customized gadgets and procedures are detailed in Figure S1.

### Electric Measurements and Approach Curves

CV and EIS were recorded using a Gamry Reference 600 potentiostat. The electric potential was applied through two Ag/AgCl wires, with reference/counter electrode leads (RE/CE) inside the pipet as ground and another Ag/AgCl as the working electrode (WE) in the outside solution. These arguably ideally nonpolarizable electrodes are adequate to support the nanoamps current in this study (negligible potential drift over ca. 8 h). For approach curves, the Ag/AgCl wire inside the polymer@NP was connected to the WE lead controlled by a CHI 900 bipotentiostat. The polymer@NP was mounted on a customized holder to have a 45° angle between the pipet capillary stem and a coverslip with a droplet of Li salt VEC solution on its surface. A z-piezo position controller (PI, E727) was used for precise control during the approach at a step size of either 10 or 20 nm.

## Results and Discussion

### Polymerization Routes and Nanotip Characterization

As shown in Scheme 1(a), two routes are designed to perform polymerization inside one NP or a bundle of NPs in one batch. route 1 allows for in situ monitoring of the conductivity changes by using the sample Li salt solution inside and outside. The NPs will be embedded in solids after polymerization and can no longer be removed as individual scanning nanoprobes. In route 2, standalone exposed nanotips are synthesized by loading only the sample solution inside (without an exterior solution). The drawback is that the reaction cannot be monitored by conductivity in real time during polymerization. Optical images of polymer@NPs from route 2 in (b) clearly show the transition from colorless to light yellow (images from route 1 NPs in Figure S1), indicating the polymerization consistent with the observation in bulk synthesis.^19^ The molecular structures of lithium salt, Li-MTFSI for a single-ion-conductor (SIC) polymerization and Li-TFSI for dual-ion-conductor (DIC) polymerization, are sketched in (c), with the corresponding radical-initiated reaction schemes in Figure S2.

**Scheme 1:**
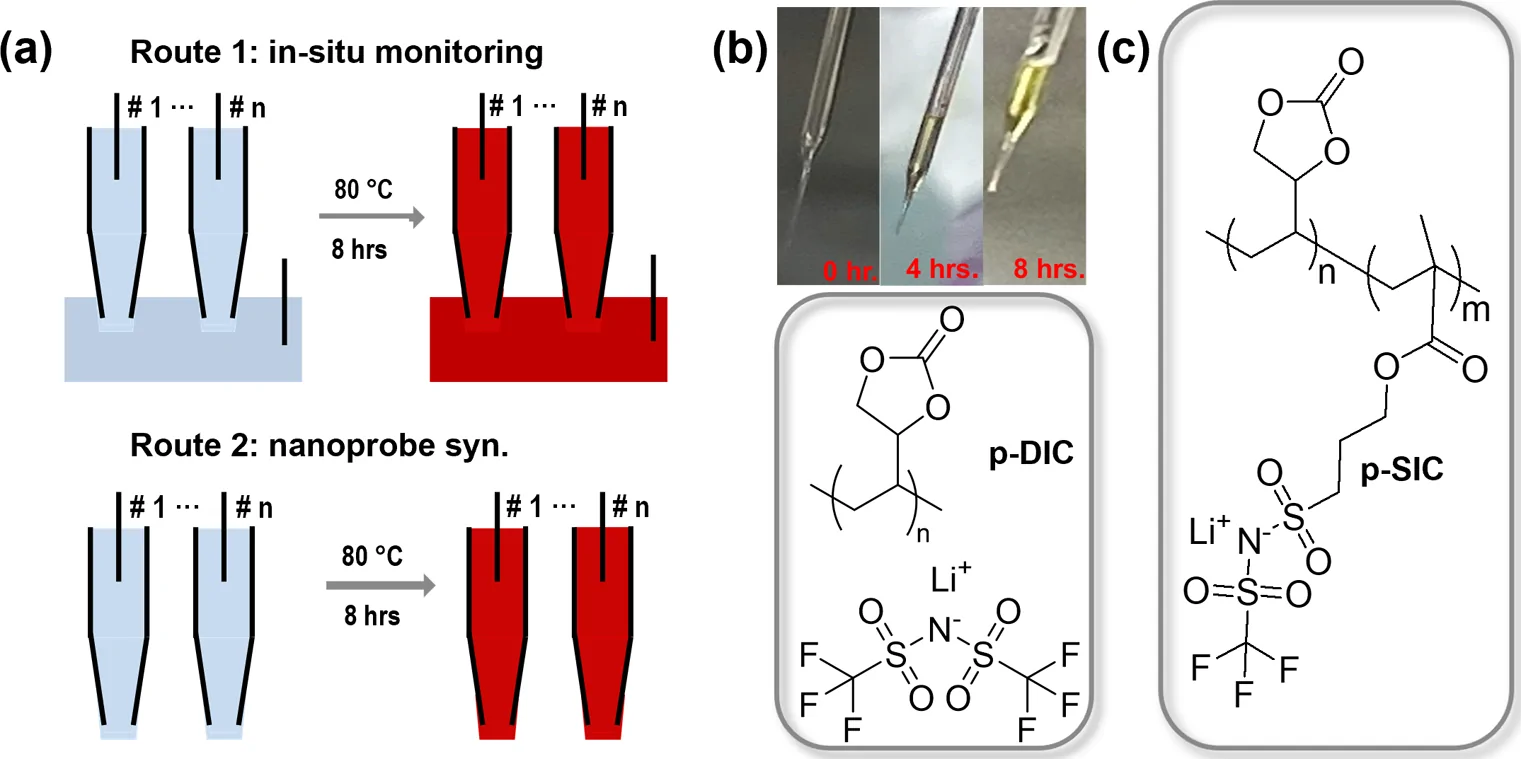
Design, Optical Images, and Molecular Structures for the Polymerization Inside Individual Conical Nanopipettes^a^

a (a) NP(s)-in-bulk setup for in situ monitoring (route 1) and nanotip synthesis (route 2). The blue fill represents Li salt dissolved in VEC with AIBN initiator prior to polymerization, i.e., the monomer solution, and the red represents polymerized solid electrolytes. The number of NPs can be 1 or up to 3–6 bundled together per trial. Not drawn to scale. (b) Time-lapse optical images of a single NP during SIC polymerization with heating time (0, 4, and 8 h). The magnification of those images is slightly different. (c) Chemical structures of p-SIC (right, from Li-MTFSI) and p-DIC (left, from Li-TFSI).

The polymer nanoprobes are characterized by SEM combined with energy-dispersive X-ray spectroscopy (EDX). Representative results from p-SIC@NPs with radii ranging from ∼50 nm to ∼1.5 μm are shown in Figure 1. The SEM images reveal slight convex meniscuses in the nanotip region in the wide range of sizes tested (a–c), which is visually different in morphology from the hollow nanotips shown in Figure S3. EDX analysis (Figure 1d) in the tip region reveals N, S, F, and C elements from the organic solid, confirming a convex p-SIC filling. As controls, the signals of organics, especially those of S and F, are absent in regions far from the nanotip or in empty NPs (not shown). It is noteworthy that maintaining high vacuum while detecting the organics during SEM/EDX analysis suggests negligible VEC evaporation and thus complete polymerization at the nanotip region. Further, continuous p-SIC filling inside, critical for conductivity measurements, is highlighted by the representative features inside a broken tip in the inset of Figure 1a. Calculated with a 9° half cone angle and at a 2 μm cross-section radius, the continuous uniform filling depth is about 13 μm (2 μm/tan(9°)). Since the electric field is the strongest at the most restricted conical nanotip under an applied potential,^47–49^ this conservative estimate of the effective volume of polymerization strongly attests to the polymer@nanotip as the transport-limiting region/step. In other words, the cross-section increases with the distance from the nanotip, which causes the impedance/resistance to decrease. Even if there were nonuniform polymer filling or variations in the total filled volume or length, the impact on the measured current or conductivity would be insignificant. Overall, SEM and EDX analyses confirm a convex p-SIC meniscus defined by the nanotip and pave the way for fundamental IT studies under nanoconfinements and as scanning nanoprobes.

**Figure 1:**
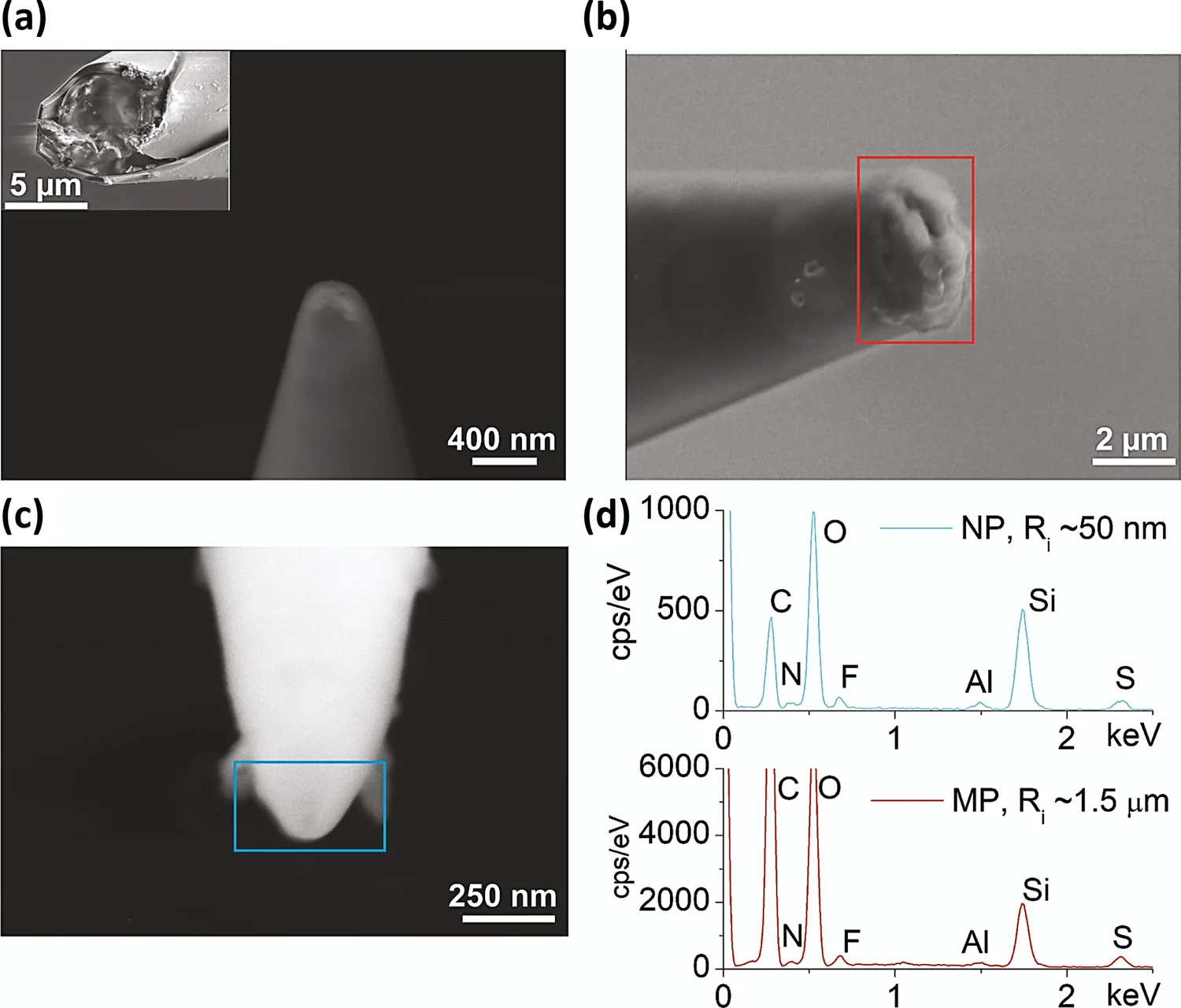
SEM and EDX analysis of p-SIC-filled nano/microsized pipettes. (a) p-SIC, *r*_*i*_ ≈ 100 nm. Inset: interior from a broken tip. (b) p-SIC, *r*_*i*_ ≈ 1.5 μm. (c) p-SIC, *r*_*i*_ ≈ 50 nm. (d) EDX spectra of the tip area of the nanopipette in panel (c) and micropipette in panel (b). Signals of C, N, S, and F elements from the tip region confirm the p-SIC filling.

### Standardization of Monomer and Polymer Conductivity and In Situ Monitoring

The ionic conductivity of Li salt in VEC as monomers is first determined to be 5.1 × 10^–4^ S/cm for Li-MTFSI and 3.6 × 10^–3^ S/cm for Li-TFSI at room temperature using eq 1, by normalizing the resistance/conductance from 1 M KCl solution as a standard with the same NPs via CV measurements (Table S1). This value is consistent from pipettes ranging from micrometers to tens of nanometers and agrees with those from bulk BDS analysis (8.5 × 10^–4^ S/cm for Li-MTFSI and 4.4 × 10^–3^ S/cm for Li-TFSI).^19^ The small difference is deemed acceptable considering the differences in samples and tools used, as further discussed in Consistency and Reproducibility by Statistical Analysis. Establishing the Li salt-VEC as an internal standard allows for the correlation of ionic conductivity changes with the progression of polymerization in individual NPs using eq 2

$$\sigma_{p-SIC}=\frac{G_{p-SIC}}{G_{\lim TFSI/VEC}}\times \sigma_{\lim TFSI/VEC}$$ 2

in which σ_*p*–*SIC*_(or σ_*p*–*DIC*_) characterizes different extents of polymerization and conductance (*S*) values (*G*_*p*–*SIC*_, *G*_*LiMTFSi*/*VEC*_, or DIC counterparts) are measured experimentally, e.g., from the slope of *I–V* curves collected at different time points during the reaction. Since geometric factors in eq 1 are canceled out, there is no need for SEM imaging or standardizing every NP with KCl conductivity, a strategy that makes the quantification otherwise unfeasible and drastically simplifies the operation.

Representative *I–V* curves during SIC polymerization and results from two NPs are summarized in Figure 2 to demonstrate the key features and general trends. Unlike aqueous electrolytes in conical nanopores displaying prominent ion current rectification (ICR) and pinched hysteretic *I–V* loops resulting from the electrostatic effects of surface charges,^50,51^ the current responses are largely linearly ohmic, suggesting insignificant EDL or surface effects under a high ionic strength (1 M) and organic solvent. The conductance (*G*) values are calculated from the slope of the *I–V* curve at 100 mV/s in the linear range, e.g., ±0.2 V, which remain consistent over different scan rates (Figure S4). It is worth mentioning that there is no significant difference in the *I–V* responses between freshly prepared and used Ag/AgCl quasireference electrodes as shown in Figure S5. This is attributed to the surface activity/concentration of Ag^+^ (as AgCl) and Ag^0^, both in the solid state, being unity and their limited solubility in VEC. Additionally, negligible changes in current are observed when using Pt vs Ag wires, further supporting that these low current nanotip-limited measurements are not sensitive to whether polarizable or nonpolarizable electrodes are used.

**Figure 2:**
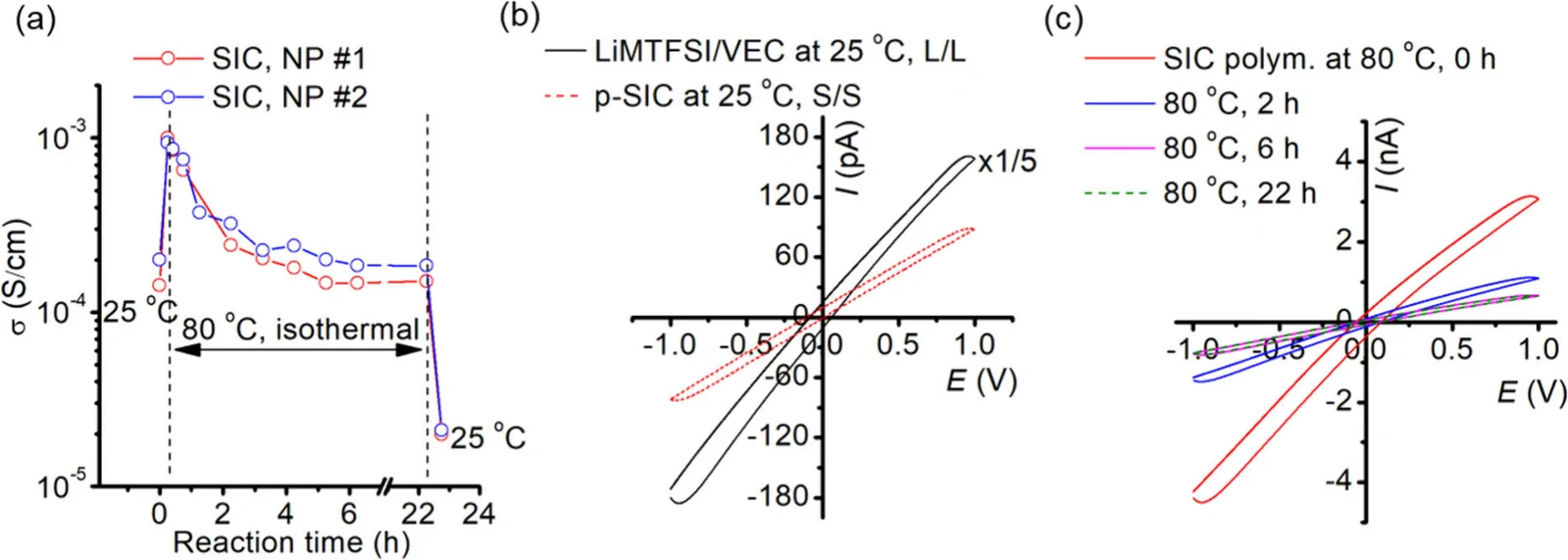
Polymerization inside single NPs monitored by CV starting from 10 mol % LiMTFSI/VEC (SIC). (a) Evolution of ionic conductivity from two NPs of *r*_*i*_ ≈ 100 nm as a function of polymerization reaction time. Time zero represents liquid–liquid (L/L: inside/outside across the nanotip) that transits to solid–solid (S/S). (b) *I–V* curves at 25 °C before and after polymerization. The current represented by the black curve was divided by a factor of 5 to be on the same scale as the red curve. (c) Representative *I–V* curves at 80 °C during polymerization. The 0 h data represents the start of polymerization at 80 °C when monomer salt should be dominant. The scan rate of CV was 100 mV/s.

With the temperature rising to 80 °C, the ionic conductivity increases by about 1 order of magnitude in the two trials shown in Figure 2a (1.0 × 10^–3^ S/cm and 9.4 × 10^–4^ S/cm for NP_1_ and NP_2_, respectively, from 1.4 × 10^–4^ S/cm and 2.0 × 10^–4^ S/cm at room temperature). It then drops gradually over 4 h during polymerization and stabilizes to 10^–4^ S/cm (1.5 × 10^–4^ S/cm and 1.9 × 10^–4^ S/cm for NP_1_ and NP_2_, respectively) with little to no changes thereafter. After cooling to room temperature, the ionic conductivity dropped to 10^–5^ S/cm (2.0 × 10^–5^ S/cm for NP_1_ and 2.1 × 10^–5^ S/cm for NP_2_), 1 order of magnitude lower than for the original Li salt-VEC monomers. An initial increase in Li salt conductivity with heating results from the decrease in viscosity and the increase in ion mobility. At elevated temperatures such as 80 °C, an increase in AIBN radicals accelerates polymer chain propagation. The LiMTFSI monomers are incorporated into the p-VEC matrix, forming a polymer framework which decreases the ion mobility, especially for anions, and thus the measured ionic conductivity. The stabilized ionic conductivity at 10^–4^ S/cm after heating for 4 h at 80 °C suggests the completion of the polymerization reaction, in good agreement with bulk synthesis (Figure S6).

While the DC conductivity interpreted from the linear ohmic slopes of the *I–V* curves is independent of the scan rate (υ), the *I–V* curves also display measurable capacitive loops which increase at higher scan rates (Figure S4). In principle, the capacitance of the interfaces at the nanotip can be extracted from the current gap between the forward and backward current branches at different scan rates (υ) or EIS. Unfortunately, Faradaic shielding and connection management are very challenging inside a glovebox for frequency-domain measurements, especially for the very low signal (sub)­nA current. From the CVs at different scan rates (Figure S4) and preliminary EIS results (Figure S7), in comparison to the results from a 0.5 GΩ solid-state resistor for estimating the stray capacitance of the measurement setup, a semiquantitative estimate of the capacitance is in the range of tens to hundreds of pF. Further optimization is underway and will be discussed in a follow-up report. We do not anticipate that other interfaces, for example, at the contacts with the two Ag/AgCl wires, would affect our measurements because the nA current can be easily supported by the facile Ag^+^/Ag reactions. This argument is supported strongly by the overlapping EIS spectra under DC biases of 0 V and ± 0.5 V: without (0 V) and with opposite (±0.5 V) polarization of either electrode/electrolyte interface made no detectable difference. It is also worth mentioning that the *I–V* curves are provided in Figure 2 to demonstrate the low noise level and thus the high signal/noise ratio of the conductivity analysis.

### Consistency and Reproducibility by Statistical Analysis

The means of ionic conductivity at room temperature (25 °C) from the Gaussian fitting of the histograms in Figure 3 are (3.0 ± 1.1) × 10^–5^ S/cm for p-SIC and (6.0 ± 2.5) × 10^–4^ S/cm for p-DIC. The values are consistent within experimental errors with those by BDS analysis of samples from bulk synthesis, 1.4 × 10^–5^ S/cm for p-SIC and 7.5 × 10^–4^ S/cm for p-DIC. The results demonstrate the successful polymerization of two different materials systems, p-SIC and p-DIC. The agreement with the bulk conductivity values is critical in establishing the nanopipette platform as a reliable and quantitative method for measuring ionic conductivity. Such consistency also indicates that nanoscale confinement, down to about 100 nm, does not have significant impacts on the ion transport of these polymer electrolytes. Interestingly, the 50 nm data appear more scattered and several have higher conductivity. The iterative G-test (Tables S2–S4) rejects only one outlier (and three if both 100 and 50 nm data are combined). As compared in Figure S8, the 50 nm data cannot be described by Gaussian fitting. Our hypothesis is that the nanoconfinement needs to approach the phase domains in those polymer systems, which along with other related material properties are under further investigation and are beyond the scope of this report.^41,42,52^ Generally speaking, the distribution of the measured conductivity is first attributed to device-to-device variability arising from the uncertainties associated with the fabrication process involving thermal melting and mechanical pulling as discussed in the Experimental Section. The other possible contributor is dust or another impurity/contaminant at the nanotip given these measurements were performed inside a glovebox but not in a cleanroom setting. It may be worth mentioning that order-one variability, i.e., differences in conductivity within the same order of magnitude, is generally less significant for polymer electrolytes where variability in size (molecular weight range) and phase domain etc. often plays more dominant roles.

**Figure 3:**
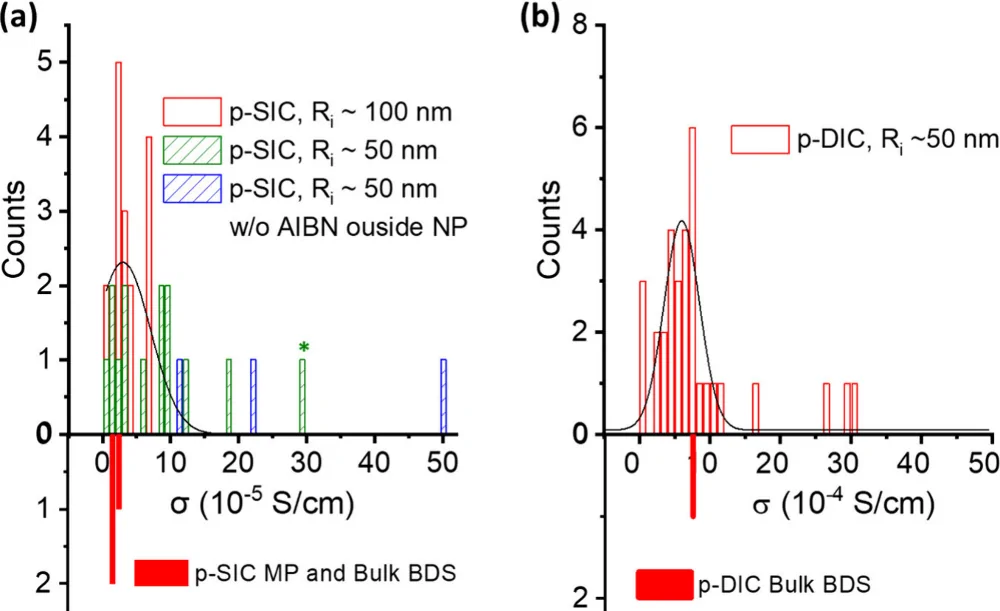
Statistical analysis of the ionic conductivity of polymer@NPs. (a) Histogram from 38 p-SIC@NPs in three groups, *R_i_* ≈ 100 nm (#20), *R_i_* ≈ 50 nm (#15), and a control group (#3) without radical initiator AIBN in the outside solution. The bottom panel (counts in fourth quadrant) provides p-SIC data from BDS analysis of a bulk film and two p-SIC’s polymerized in micrometer-sized (∼4 μm) pipettes for comparison. A Gaussian fitting curve was created from the 20 data points from the *R_i_* ≈ 100 nm group. The asterisk indicates the outlier identified by a G-test when analyzed within the 50 nm NPs data set. (b) Histogram from 32 p-DIC@NPs (*r*_*i*_ ≈ 50 nm), in comparison with bulk p-DIC data from BDS analysis of a bulk film.

The polymers under nanotip confinement are further characterized by thermal activation energy. Each data series in Figure 4 was recorded from a polymer@NP at discrete temperatures, starting from room temperature up to 80 °C. The temperature was stabilized for about 15 min, after which repeated CV curves overlap, from which consistent conductivity was calculated based on at least two different scan rates, mostly 100 mV/s and 500 mV/s. Representative original *I–V* data are provided in Figure S9. Extracted from the slopes of the linear fitting following the Arrhenius relationship, the averages of p-SIC@NPs and p-DIC@NPs are determined to be 36 ± 7 kJ/mol and 20 ± 4 kJ/mol, respectively, after complete polymerization (S/S systems, panels a and b). The results are in excellent agreement with bulk films (solid red symbols),^19,33^ though our nanopipette measurements were restricted from room temperature up to 80 °C due to the lack of cooling capability to capture the Vogel–Fulcher–Tamman (VFT) dependence.^19^

**Figure 4:**
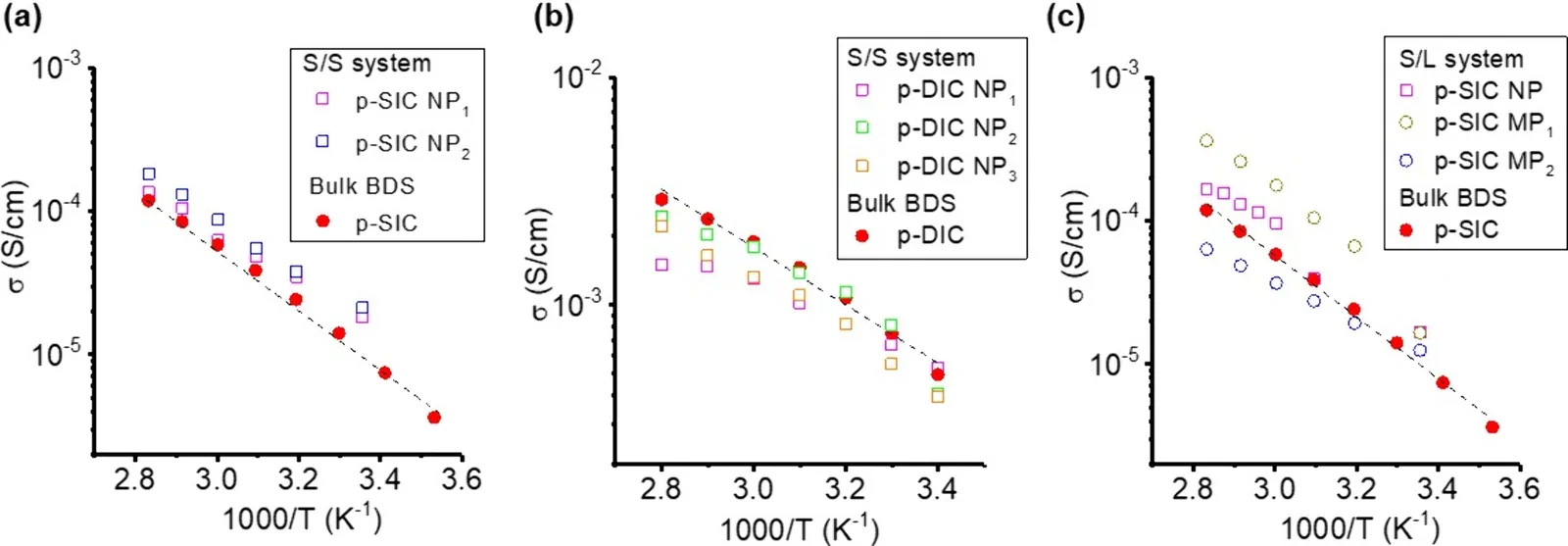
Temperature dependence of ionic conductivity of (a) p-SIC@NPs synthesized via route 1 (solid inside/solid outside, S/S), (b) p-DIC@NPs synthesized via route 1 (S/S), and (c) p-SIC@NPs synthesized via route 2 and dipped into 1 M LiMTFSI in VEC (solid inside/liquid outside, S/L). Hollow symbols represent replicated data series from different nanometer–micrometer-sized pipettes. Data from the slope of the 100 mV/s *I–V* curve in the linear range, e.g., ±0.2 V. Solid symbols are BDS data from bulk p-SIC films in a previous report.^19^

To evaluate the usability of the polymer@NPs as nanoprobes, p-SIC@NPs synthesized via route 2 are dipped into a liquid electrolyte solution such as the Li salt monomers, as prototypes for probing IT across a solid–liquid interface. With comparable nanotip sizes (*r*_*i*_ ≈ 100 nm, magenta hollow symbols in Figure 4a, the S/S), this S/L system exhibits a similar ionic conductivity of 10^–5^ S/cm at room temperature (Figure 4c). This observation suggests that the IT is governed by the SSE portion of p-SIC@NP due to its much lower conductivity compared to that of liquid LiMTFSI/VEC (10^–4^ S/cm). Similarly, excluding radical initiator AIBN in the exterior solution during polymerization made no distinguishable differences in the measured ionic conductivity (Figure 3a), which would reduce the extent of polymerization and cause the exterior solution to remain more liquid-like (S/S versus S/L). These results further confirm that the SSE in the nanotip is the transport-limited region due to its much lower conductivity compared to that of liquid electrolyte solution counterparts.

### Approach Curves of the Polymer Nanoprobes

Controlling the nanotip–substrate distance is the first and arguably the most critical step in establishing nanoprobe imaging and analysis capabilities. Different from the widely adopted vertical approach in SICM, we use a tilted alignment so that the nanotip is driven toward the surface at a non-90° angle as illustrated in Figure 5a.^53^ This design takes advantage of the lateral flexibility of the sharp-thin nanotip and reduces the risk of tip crushing. When the polymer nanotip is in the bulk far from the surface, steady-state current (*I*_0_, nA) is governed by eq 3, including both nanopore resistance (*R*_*p*_, Ω) and access resistance (*R*_*a*_, Ω) modified from eq 1,

$$I_{0}=\frac{V_{0}}{R_{p}+R_{a}\left(d\to \infty \right)}=-\frac{V_{0}}{\frac{1}{\sigma_{p}\pi r_{i}\tan\theta }+\frac{1}{4\sigma_{m}r_{i}}}$$ ; 3

where σ_*p*_ and σ_*m*_ are the ionic conductivities (S/cm) of the polymer@NP and the exterior matrix or point of interest, respectively, and *V*_0_ is the applied potential (V). Note that we use σ_*m*_ of 1 M LiTFSI in VEC under the ambient environment here and that the monomer used for polymerization as a prototype for method development can obviously be replaced with other samples of interest in future applications. As the p-DIC@NP approaches the substrate surface, the ion current *I*(*d*) decreases due to the blocking of IT by an insulating surface referred to as negative feedback. Experimentally, the approach is stopped when *I*(*d*) decreases up to about 50% of *I*_0_. This contrasts sharply with common SICM practice where only about a 1–10% current decrease is used to avoid tip damage. Additional data points in approach curves not only improve the curve-fitting quality but also enable closer proximity toward resolving finer structures. The range of piecewise fitting and rejection of outliers are based on deviations >3*N* of *I*_0_, where *N* is the standard deviation representing the noise level.

**Figure 5:**
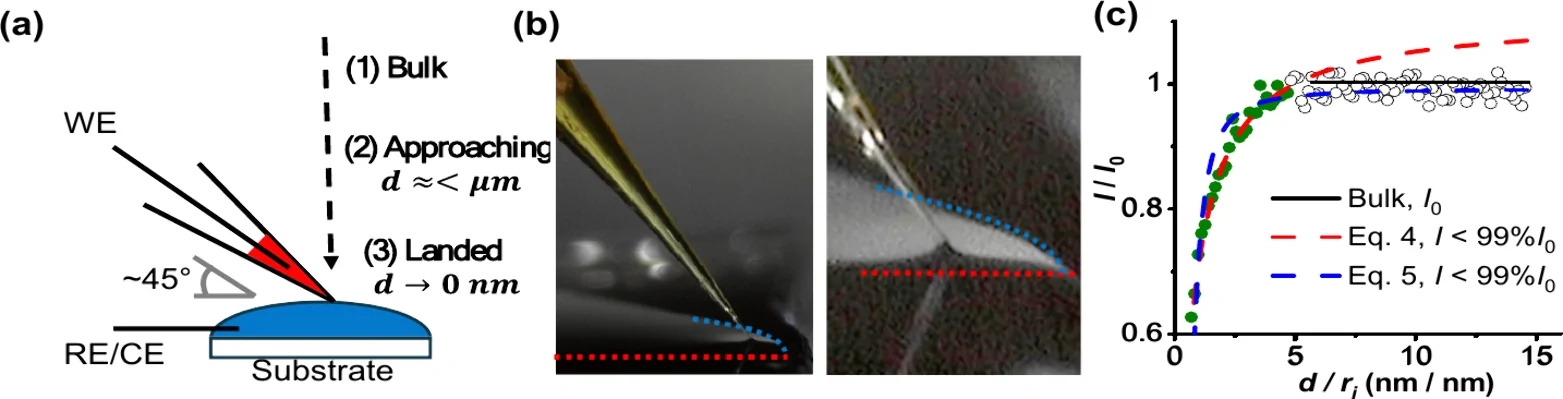
Nonvertical approaching and tip–substrate distance (*d*) analysis with a p-DIC@NP. (a) Schematics of a 45° alignment between the nanopipette stem and an insulating glass substrate under a 10 mol % LiTFSI/VEC (1 M) droplet. The dashed arrow indicates the approach direction. (b) Side-view optical images of a p-DIC@NP near the substrate (left), with a zoomed-in view (right). The blue and red dashed lines illustrate the droplet surface and the substrate, respectively, where the mirror image from the bottom side can be visualized. (c) Representative approach curve from a 70-nm-radius p-DIC NP. The current was normalized by the steady-state current when the nanotip is in the bulk, i.e., more than micrometers away from the substrate. The inner radius *r*_*i*_ was determined from *I*_0_ = −2.3 nA (open circles, mean value shown as a black solid line) using eq 3. Solid points were used for fitting with eqs 4 and 5. The piezo speed was 100 nm/s at a step size of 10 nm (raw data in Figure S10a; additional examples and fitting details in Figures S10 and S11; fitting statistics in Table S5).

After incorporating σ_*p*_ and σ_*m*_ into the universally adopted resistance expression in the SICM literature,^11,38,54,55^ the current as a function of tip-to-substrate distance (*d*) is expressed in eq 4

$$I\left(d\right)=\frac{V_{0}}{R_{p}+R_{a}}≅I_{0}{\left(c+\frac{\frac{3}{2}\ln\left(\frac{r_{o}}{r_{i}}\right)r_{i}\tan\theta }{\frac{\sigma_{m}}{\sigma_{p}}d}\right)}^{-1}\;=I_{0}{\left(c+\frac{\frac{3}{2}\ln\left(\frac{r_{o}}{r_{i}}\right)r_{i}\tan\theta }{ad}\right)}^{-1}$$ 4

where $R_{a}=\frac{1}{4\sigma_{m}r_{i}}+\frac{\frac{3}{2}\ln\left(\frac{r_{o}}{r_{i}}\right)}{\sigma_{m}\pi d}\approx \frac{1}{4\sigma_{m}r_{i}}$ when *d* → ∞ (eq 3). When *d* is smaller, the tip is near the surface and the first term becomes insignificant (eq 4). The parameter *c* should equal 1 in theory. Because the initial tip position (*Z*_0_, nm) varies experimentally, *d* or *Z*_0_ is determined from fitting the approach curve, with travel distance *Z* (nm) determined by piezo speed υ (nm/s) and time (*d* = *Z*_0_ – *Z* = *Z*_0_ – *υt*).

Under the circumstance that σ_*p*_ is found to be much smaller than σ_*m*_, σ_*m*_/σ_*p*_ > 1, eq 4 fails to fit the experimental approach curve even qualitatively (not shown). Fitting parameter *a* is introduced to test the hypothesis of unforeseen changes in σ_*p*_ (σ_*m*_ is a bulk value independent of NPs). Another fitting parameter *c* is used to improve fitting, accounting for possible drift and other experimental uncertainties. The best fitting quality, a representative one in Figure 5c and more examples in Figures S10 and S11, is achieved with σ_*m*_/σ_*p*_ in the range of ∼0.1, which suggest that σ_*p*_ would be about 10 times higher than σ_*m*_. While a higher σ_*p*_ is very favorable for applications, we are unaware of any established mechanism to support this fitting outcome. Parameter *c* also deviates from 1 by 10–40% less, which is beyond known hardware drift or experimental uncertainties. With the data range for fitting set from 99%*I*_0_ to the last point collected (about 60%*I*_0_), significant deviation from the experimental current occurs at large *d*, about 5*r*_*i*_, where the current should converge with *I*_0_. Therefore, despite the high fitting quality within the current decrease portion, the lack of valid physicochemical meanings in the fitted values requires mechanistic considerations.

The interface between the polymer nanotip and the liquid electrolyte can be described by a capacitance term. When the polymer nanotip is near the substrate, the expression based on the charging of a “plate” capacitor, at a first-level approximation, is incorporated into the approach current expression in eq 5

$$I\left(d\right)=I_{0}{\left(1+\frac{\frac{3}{2}\ln\left(\frac{r_{o}}{r_{i}}\right)r_{i}\tan\theta }{\frac{\sigma_{m}}{\sigma_{p}}d}\right)}^{-1}-V\frac{\varepsilon_{0}\varepsilon A}{d^{2}}\upsilon =I_{0}{\left(1+\frac{\frac{3}{2}\ln\left(\frac{r_{o}}{r_{i}}\right)r_{i}\tan\theta }{\frac{\sigma_{m}}{\sigma_{p}}d}\right)}^{-1}-\frac{b}{d^{2}}$$ 5

where *V* is the potential (V) applied during the approach; υ is the speed of the piezoscanner (nm/s); *A* is the effective area (nm^2^) of the exposed polymer on nanotip; and *ε*_0_ and *ε* are the dielectric constants of the vacuum and matrix, respectively. Derivation details are in the Experimental Section in the SI. These constants are combined into a single fitting parameter *b*. Mechanistically, those ions involved in the capacitive charging would not contribute to the DC transport current *I*(*d*) and need to be subtracted from the first term (which is based on geometric resistance/conductance).

The capacitance expression passes the first test in decreasing sharply at larger *d*, where the current returns to *I*_0_. Fitting of the approach curve using eq 5 in Figure 5c, notably using the measured σ_*p*_ and σ_*m*_, shows much improved agreement with the experimental data in the whole range. The next evaluation is the hundreds of pF capacitance calculated from the fitted *b* values (Table S5; $C=\frac{b}{V\upsilon d}$ ), which is in excellent agreement with the CV and EIS characterizations discussed in earlier sections. It is worth mentioning that this interfacial capacitance cannot be differentiated from stray capacitance in our current measurement setup, which is under further study and would not affect the major findings in this report. Figure S10 and Figure S11 provide additional examples showing reproducibility using the same NP repeatedly and differently sized NPs. All results confirm the efficacy and improved fitting quality using eq 5. It is noteworthy that both eq 5 and eq 4 generate about the same *d* or *Z*_0_ (less than *r*_*i*_, Table S5). Although the *d* or *Z*_0_ is insensitive to the curve shape near the upper threshold of the data range (99%–90%, Table S5), the deviations indicate effects such as the plate capacitance approximation, differences in effective versus geometric area, and more critically changes in σ_*p*_, all of which require further study. The slight variation with a lower threshold by about 20–50 nm (Table S6) again attests to the significance of the closer approach of the nanotip to the substrate through the tilted alignment. Interestingly, one example shows that the current stops changing while the piezo motor continues to push the NP forward, while *d* decreases to zero and even becomes negative (an artifact reflecting that time increases or the movement of the motor rather than the nanotip). The results can be explained by the nanotip landing at the substrate. Because of the 45° approaching angle, the quartz wall serves as a constant separator under the sideways configuration after the initial contacts. Further advances by the piezomotor only push the stem closer, while the nanotip–substrate distance and thus the IT current remain unchanged. Timely retrieval of the NPs will be critical to avoid tip damage. The recovery of the *I*_0_ indicates an intact NP, and an increase in *I*_0_ suggests (partial) breaking of the nanotip. While optimization is underway and more systematic studies are needed, especially for the capacitance expression, the high-quality approach data and successful curve fitting validate the functionality of the polymer@NP probes and demonstrate their capability and potential for noncontact and contact mode analysis of IT at polymer SSE/substrate interfaces.

## Conclusions

We present the successful derivatization of individual conical nanopipettes via the radical-initiated thermal polymerization of lithium electrolytes. Two synthetic setups are developed: one for in situ monitoring of the ionic conductivity changes associated with polymerization and another producing standalone polymer nanotips. The strategy and devices used to perform polymerization reactions using either a single nanopipette or a bundle of them inside a glovebox environment are shown to be effective using different material systems, specifically single and dual ion conducting polymer solid-state electrolytes. The reactions are characterized by an initial increase in the ionic conductivity with an increase in temperature, followed by conductivity decreases by 1 order of magnitude indicating the propagation of polymer chains. At room temperature, p-SIC@NP and P-DIC@NP have ionic conductivities of 10^–5^ S/cm and 10^–4^ S/cm, respectively, both of which are consistent with standard BDS results from bulk films made from the same materials. Proof-of-principle nanoprobe measurements are demonstrated through repetitive approaching of an insulating surface under a liquid electrolyte droplet. Technical improvements such as nonvertical alignment between the nanotip and substrate reduce the risk of tip breaking and allow for closer approaches. An analytic expression of the capacitance term is introduced for the effective fitting of the approach curves. Standardized polymer SSEs in individual nanoprobes lay the foundation for exploring ion transport with another material or interface at a nanoscopic interface defined by the nanotip. The developed tools and strategies are generalizable to other polymer electrolytes or target materials/interfaces.

## Supplementary Material



## Notes

Additional synthesis and measurement details; SEM images; CV and EIS results; approach curves and fitting results (PDF)

## §

S.R.P.: Department of Chemistry, Brescia University, Owensboro, Kentucky 42301, United States
