# Supplementary material for: Development of Ionic Polymer-Functionalized Single Nanopipette Probes
Source: Anal Chem. 2026 Aug 29;98(36):26767–76. doi: 10.1021/acs.analchem.6c03481 (PMC13584631; doi:10.1021/acs.analchem.6c03481)
Supplement: Supplementary file 1 [file ac6c03481_si_001.pdf]

Supporting Information

Development of Ionic Polymer Functionalized Single Nanopipette Probes

Kan Tang[†‡], Surendra R Puri[†‡], Milton Ahamed,[†] Ruoyu Yang[†], Dipak Baram[†], Ji-young Ock,[#] Xi Chelsea Chen, [#] Gangli Wang[†,]*

[†]Department of Chemistry, Georgia State University, Atlanta, Georgia, 30302, United States
[#] Chemical Sciences Division, Oak Ridge National Laboratory, Oak Ridge, Tennessee, 37830, United States

---

Table of Contents

Materials and Methods

List of Tables
Table S1: Ionic conductivity of Li salts and polymerized p-SIC and p-DIC.
Table S2: Grubbs' test results of p-SIC NPs (R_i ~50 nm and R_i ~100 nm data combined, 35 data points).
Table S3: Grubbs' test results of p-SIC NPs (R_i ~50 nm separately, 15 data points).
Table S4: Grubbs' test results of p-DIC@NPs (R_i ~50 nm, 32 data points).
Table S5: Summary of fitting parameters for p-DIC@NPs approach curves.
Table S6: Dependence of tip-substrate distance on fitting parameters.

List of Figures
Figure S1: Photos of the in-house built gadgets and operation procedures for nanopipette handling and electrical measurements.
Figure S2: AIBN initiated polymerization reactions of Li single-ion-conductor (top, p-SIC) and dual-ion-conductor (bottom, p-DIC).
Figure S3: Additional SEM images of empty nano-micro-pipettes.
Figure S4: Additional CV results at different scan rates associated with SIC@NP polymerization.
Figure S5: *I-V* curve comparison for (A) fresh vs used electrode and (B) Pt vs Ag/AgCl electrodes.
Figures S6: The evolution of ionic conductivity during SIC polymerization at 80 ºC from BDS analysis.

Figure S7: Electrochemical impedance spectroscopy (EIS) analysis of (a, b) a 0.5 GΩ (R_0) and (c, d) a p-SIC solid-solid (S/S) interface under the same measurement conditions.
Figure S8: Gaussian fitting of conductivity of p-SIC NPs with (a) R_i ~100 *nm (#20)* and R_i ~50 nm *(#15)* combined; and (*b*) R_i ~50 nm only.
Figure S9: *I-V* curves of a p-SIC filled NP (R_i ~100 nm) at various temperatures.
Figure S10: Approach curves (in original current) from additional polymerized p-DIC@NPs.
Figure S11: Normalized tip current (I/I_0) of p-DIC NPs with various tip sizes (r_i) as a function of tip-substrate distance (*d*).

Materials and Methods

Materials and Chemicals

For polymerization of single-ion-conductor (p-SIC) and dual-ion-conductor (p-DIC): >98.00 % 4-Vinyl-1,3-dioxolan-2-one (common name: vinyl ethylene carbonate, VEC, TCI America) dried over molecular sieves, 94.00% Lithium sulfonyl-(trifluoromethane sulfonyl)imide methacrylate (LiMTFSI, Specific Polymers), >98.00% Lithium bis(trifluoromethanesulfonyl) imide (LiTFSI, TCI America), 98.00% Azobis (isobutyronitrile) (AIBN, Sigma-Aldrich) recrystallized in methanol. For electrochemical measurements: 99.90 % Ag wires, dia 0.25 mm (Sigma Aldrich), For Ag wire coating: 97 % $FeCl_3$ (Sigma Aldrich). Capillaries for pipette fabrication: Quartz (O.D.: 1.0 mm, I.D.: 0.70 mm, 7.5 cm length, with filament, QF100-70-7.5) and borosilicate capillaries (O.D.: 1.0 mm, I.D.: 0.75 mm, 15 cm length, no filament, B100-75-15) (Sutter Instrument Company).

Instruments and Measurements

All electrochemical measurements of NP-in-tubes and NPs were performed using a Gamry Reference 600 potentiostat (Gamry Co.) inside the glovebox (VTI Super 1220/750/900, Vacuum technology instruments Inc.). The current was sampled at a voltage resolution of 1 mV per point in cyclic voltammetry while monitoring the polymerization of DIC and SIC. The electrical bias was applied through two Ag/AgCl wires, with RE/CE inside and WE outside the pipette in contact with solid or liquid electrolyte on the bottom of the Eppendorf tube. Electrochemical impedance of the NP-in-tube devices was carried out using a Gamry Interface 1010E (Gamry Co.) in the Faraday cage within 1 h of the devices transferred out from the glovebox. Digital Piezo Controller (PI E-727, Physik Instrumente L.P.) was used to acquire approach curve where process was visualized via Dino-lite camera with polarizer (AM4113ZTL, Dunwell Tech Inc.) and electrical bias was applied through CHI 900 potentiostat (CH Instruments Inc.).

Pipette fabrication

Nanopipettes (NPs) and micropipettes were fabricated from quartz capillaries (QF100-70-7.5) and (B100-75-15) using a P-2000 laser puller (Sutter Instrument Co.). The pulling parameters for nanopipettes with ~r_i of 50 nm are: line 1, Heat: 700, Filament: 4, Velocity: 55, Delay:180, Pull: 80 and line 2, Heat: 700, Filament: 4, Velocity: 60, Delay:150, Pull: 150; for nanopipettes with ~r_i of 100 nm are: Heat: 555, Filament: 3, Velocity:50, Delay:130, Pull: 90; for micropipettes with ~r_i of 1.5 µm are: Heat: 350, Filament: 4, Velocity:30, Delay: 200, Pull: 0.

In-Situ Polymerization of NP-in-Tubes and Single NPs

The pulled pipettes were placed into pipette holders modified from Eppendorf tubes. The setup was washed thoroughly with DI water and dried in an oven before being transferred into a nitrogen-filled glovebox. If the pipettes were tested in 1M KCl, in addition to ID water rinsing, multiple CV scans in DI water were performed until the current reach a low threshold as previously reported.[1,2] Inside the glovebox, typically, VEC monomer (4.38 mmol, 0.5 g) was mixed with 0.438 mmol of SIC (LiMTFSI, 0.152 g) or DIC (LiTFSI, 0.125g) and 43.8 µM of AIBN initiator. The mixture was vigorously vortexed to form a clear solution. The pipettes were backfilled with SIC or DIC mixture and centrifuged at 4500 rpm for 2 min. For the in-situ polymerization in an NP-in-tube setup, an SIC or DIC-filled NP was placed back into an Eppendorf tube loaded with the same mixture at the bottom. In both routes mentioned in **Error! Reference source not found.**, a 10 mol% Li salt in VEC solution with the presence of 1 mol% AIBN as an initiator was backloaded into an empty nanopipette (NP). For the polymerization in NPs for in-situ monitoring (route 1), the loaded NPs are protected by a disposable plastic pipette tip and inserted into an Eppendorf tube holder that was also filled with the same solution. For the polymerization in a standalone NP (route 2), a filled NP was secured into an empty Eppendorf tube. After reassembling the NPs, the Eppendorf tubes were inserted into a customized aluminum heating block or a heat tape for thermal polymerization that was carried out at 80 °C inside the glovebox. To improve efficiency in collecting statistically sound data, we designed a parallel 'high throughput' synthetic setup by positioning multiple loaded NPs, normally 4-6 and each with a separate electrode lead inserted from the back end, inside a small Eppendorf vial containing the same solution and a counter electrode lead.

Approach curves of p-DIC nanopipettes

10 mol% LiTFSI/VEC (DIC, 1 M) with 1 mol% AIBN initiator was back-filled into glass nanopipettes using a micro-injector inside a glovebox. An Ag/AgCl wire was inserted into nanopipettes as the working electrode. The polymerization of DIC was carried out at 80 °C overnight (~16 h). After the polymerization reaction was finished, the p-DIC@NP tip was transferred out of the glovebox and clamped onto a customized tip holder that is mounted on a z-piezo position controller. A glass slide with a droplet of 1 M LiTFSI in VEC was placed underneath the tip holder as the substrate for the tip approaching. Another Ag/AgCl wire in contact with the droplet served as both quasi-reference and counter electrode. During the tip approaching, a voltage of V_0 = -1 V was applied to the working electrode by a CHI 900 potentiostat. The resultant current

signal was recorded by the same instrument and sent to the piezo controller to synchronize with the z-position.

Scanning electron microscopy (SEM) and energy dispersive x-ray spectroscopy (EDX) Characterization

The effectiveness of the polymerization inside the nanopipettes was examined using Hitachi SU-8230 FE-SEM equipped with cold field emission (CFE) gun (Hitachi High-Technologies Corporation) coupled with an Oxford EDS detector. The stem of the polymer–nanopipette was cut to obtain 1.0-1.5 cm near tip region and mounted on a 35° inclined pin stub using carbon tape. An excess metal wire (Cu or Ag) was inserted into the nanopipette prior to polymerization reaction to ensure proper grounding by connecting polymer into carbon surface in pin stub. This is necessary to reduce charging during SEM imaging, as metal deposition was not adopted to ensure adequate elemental signal detection under EDX. The pipette was tilted an additional ~35°, giving an effective viewing angle of ~70° to obtain better tip view image. Elemental analysis was carried out using EDX by scanning a defined rectangular area on the sample. Side-view images of the pipette were obtained without tilting. High-resolution SEM images were collected at 2–5 kV to reduce charging effects, while EDS spectra were recorded at 10–15 kV. A pressure of ~10^{-4} Torr was maintained during both SEM imaging and EDS acquisition.

Derivation of the capacitive-corrected SICM approach equation

1. Incorporation of different conductivity terms into distance-dependent transport-resistance limited current

When the polymer-modified nanopipette (p-DIC@NP) is far from the substrate ($d \rightarrow \infty$), the total resistance of the system (R_{total}) is the sum of the nanopore resistance (R_p, dependent on polymer conductivity σ_p) and the access resistance (R_a, dependent on sample conductivity, σ_m). Following the established geometry for conical nanopores:

$$R_p = \frac{1}{\sigma_p r_i \pi tan\theta}$$

$$R_a(d \rightarrow \infty) = \frac{1}{4\sigma_m r_i}$$

$$R_{total} = \frac{1}{\sigma_p r_i \pi tan\theta} + \frac{1}{4\sigma_m r_i}$$

The steady-state current I_0 is then defined by Ohm's law as used in **Eq. (3)**:

$$I_0 = \frac{V}{R_{total}} = \frac{V}{\frac{1}{\sigma_p r_i \pi tan\theta} + \frac{1}{4\sigma_m r_i}}$$

As the tip approaches an insulating surface, the access resistance R_a increases due to the physical blocking of ion flux. Based on the widely adopted negative feedback model in SICM, the distance-dependent access resistance $R_a(d \to 0)$ is approximated by:

$$R_a(d \to 0) \approx \frac{1}{4\sigma_m r_i} + \frac{\ln\left(\frac{r_o}{r_i}\right)}{2\sigma_m \pi d}$$

Substituting this into the expression for $I(d)$ and normalizing by I_0, we arrive at the normalized form used for fitting in **Eq. (4)**:

$$I(d) = \frac{V_0}{R_p + R_a(d \to 0)}$$

$$\frac{I(d)}{I_0} = [c + \frac{\frac{3}{2} ln\left(\frac{r_o}{r_i}\right) r_i tan\theta}{\frac{\sigma_m}{\sigma_p} d}]^{-1}$$

In this expression, the term $a = \frac{\sigma_m}{\sigma_p}$ represents the conductivity ratio. Ideally, $c = 1$. However, as noted in the main text, this purely resistive model fails when $\sigma_p \ll \sigma_m$, necessitating the inclusion of a non-faradaic component.

2. Incorporation of a capacitive term

To account for the experimental deviations observed at close proximity, we consider the interface between the polymer nanotip and the liquid electrolyte as a plate capacitor in approximation. The capacitive current I_c is the time derivative of the charge accumulated at the interface:

$$I_c = \frac{dQ}{dt} = \frac{VdC}{dt} = V(\frac{dC}{dd} \cdot \frac{dd}{dt})$$

The capacitance C is distance-dependent as the tip-to-substrate gap d varies: $\frac{dC}{dd} = -\frac{\varepsilon\varepsilon_0 A}{d^2}$, where ε_0 is the vacuum permittivity, ε is the dielectric constant of the matrix, and A is the effective surface area of the exposed polymer. During the approach, the piezo moves the tip at a constant velocity $v = -\frac{dd}{dt}$. Combining those:

$$I_c = V\left(-\frac{\varepsilon\varepsilon_0 A}{d^2}\right)(-v) = V\frac{\varepsilon\varepsilon_0 A}{d^2} v$$

The ions contributing to the capacitive charging of the polymer interface are effectively "tapped" from the steady-state flux, reducing the measured DC current. Therefore, the capacitive term is subtracted from the resistive ion current:

$$I(d) = I_0\left(1+\frac{\frac{3}{2}\ln\left(\frac{r_o}{r_i}\right) r_i tan\theta}{\frac{\sigma_m}{\sigma_p} d}\right)^{-1} - V\frac{\varepsilon_0 \varepsilon A}{d^2}v$$

When we consolidate the physical constants into a single parameter $b = V\varepsilon_0\varepsilon A v$, resulting in **Eq. (5)**:

$$I(d) = I_0\left(1+\frac{\frac{3}{2}\ln\left(\frac{r_o}{r_i}\right) r_i tan\theta}{\frac{\sigma_m}{\sigma_p} \boldsymbol{d}}\right)^{-1} - \frac{b}{d^2}$$

To substantiate the physical validity of the fitting parameter b, first, we can estimate the distance-dependent capacitance $C(d)$ using the values obtained from **Equation (5)** in Figure 5c. The relationship is defined as:

$$C = \frac{\varepsilon\varepsilon_0 A}{d} = \frac{b}{Vvd}$$

Using the fitted parameter $b = 2.7 \pm 1.1 \times 10^3\ nA \cdot nm^2$ at a constant piezo approach velocity of $v = 100\ nm/s$, the calculated capacitance values are $C = 2.7\ nF$ at $d = 10\ nm$ and $C = 270\ pF$ at $d = 100\ nm$. Notably, these values are in excellent agreement with the experimental capacitance measured independently in CV and EIS. This quantitative consistency confirms that the deviation from the classical resistive model is indeed dominated by the capacitive charging of the polymer-electrolyte interface rather than instrumental drift or secondary faradaic processes. As discussed in the scan rate dependent CV and EIS sections in the main text, the interfacial capacitance cannot be decoupled from any stray capacitance in our measurements at this point, a topic is under further exploration and does not affect the main findings of this report.

Detailed fitting statistics are in Table S5 after the fitting results shown in Figure S11.

Table S1. Ionic conductivity of Li salts and polymerized p-SIC and p-DIC.

S.N.	Sample/Method	σ (S/cm)	Sample/Method	σ (S/cm)
1	m-SIC/Cap	5.10×10^{-4}	m-DIC/Cap	3.63×10^{-3}
2	m-SIC/BDS	8.50×10^{-4}	m-DIC/BDS	4.40×10^{-3}
3	p-SIC/BDS	1.40×10^{-5}	P-DIC/BDS	7.50×10^{-4}
4	p-SIC (Gaussian fit)	3.00×10^{-5}	p-DIC (Gaussian fit)	6.00×10^{-4}

The letter "m" for the corresponding Li salt in VEC as monomers, "p" for polymer, "Cap" for a cylindrical capillary as conductivity cell for CV measurements, BDS for broadband dielectric spectroscopy using an Alpha-A spectrometer. All results are at room temperature.

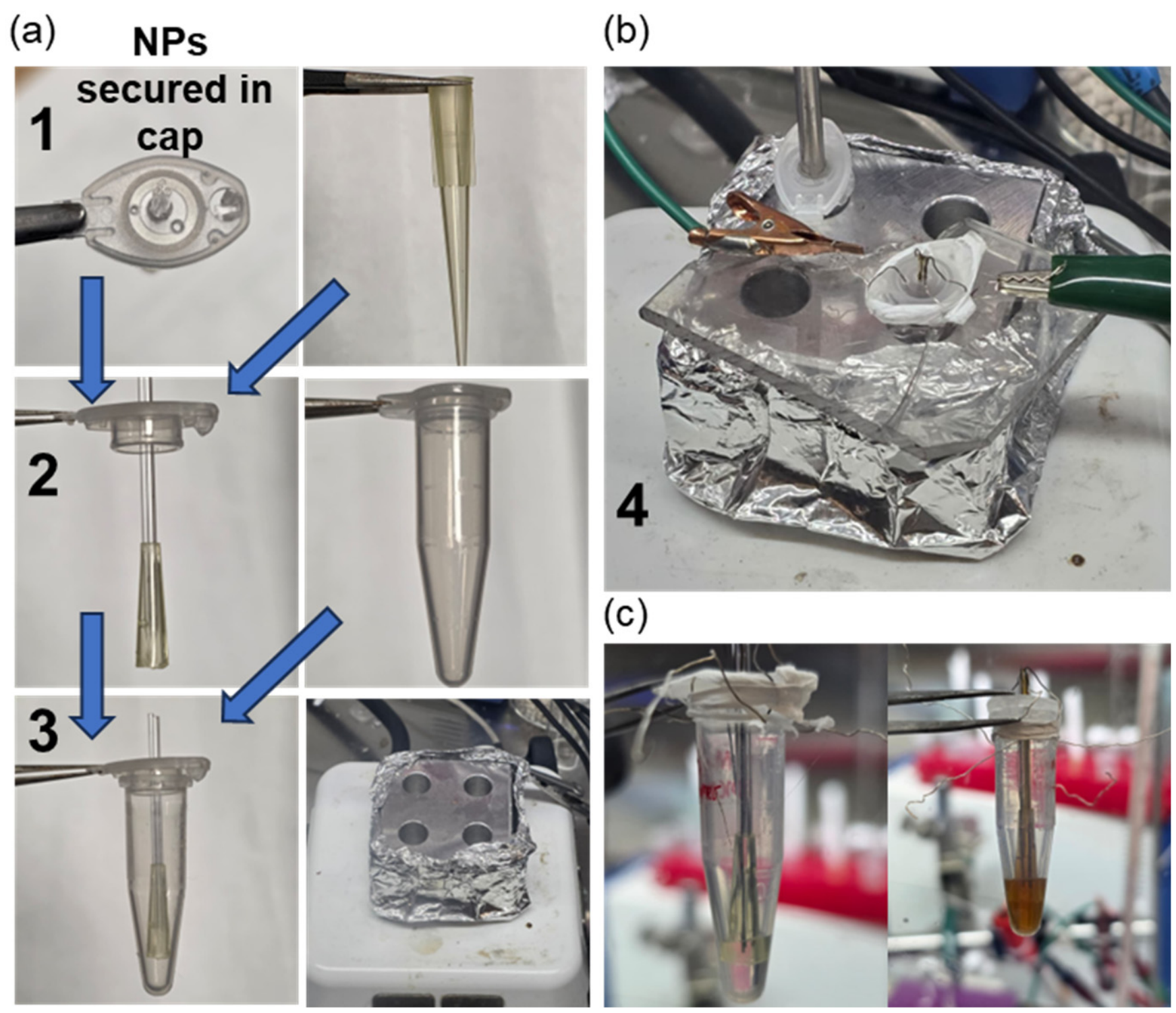

Figure S1. Photos of the in-house built gadgets and operation procedures for nanopipette handling and electrical measurements. (a) Stepwise procedure for making protective shroud around nanopipettes (NPs) secured in an Eppendorf tube as reaction holder. (b) The NPs-holder assembly in a homemade aluminum heating block for polymerization and in-situ electrochemical measurements. (c) Optical images of (SIC) monomer in an NP (left) and after polymerization, p-SIC (right). Note each NP contains one Ag wire making electrical contact inside the capillary stem; one Ag wire is inserted in the exterior solution as a common counter electrode.

Firstly, a disposable plastic pipette tip is cut so that one or a bundle of NPs can be inserted from the tip end with a tight nudge fit. The nanotips remain inside the plastic pipette. The center of the cap is drilled through so that the stems of the NPs are aligned and fixed avoiding potential damage. During heating, a thermal couple is inserted in the same type of Eppendorf tube containing the same solvent. The assembly is inserted into the heating block the same as the reaction vessel to monitor temperature in real-time. For electrochemical recording, an insulating plastic plate is used to avoid shorting the Ag wires. The bottom block is grounded with additional Aluminum foils (not shown) to shield the reaction vessel from external electrical noise.

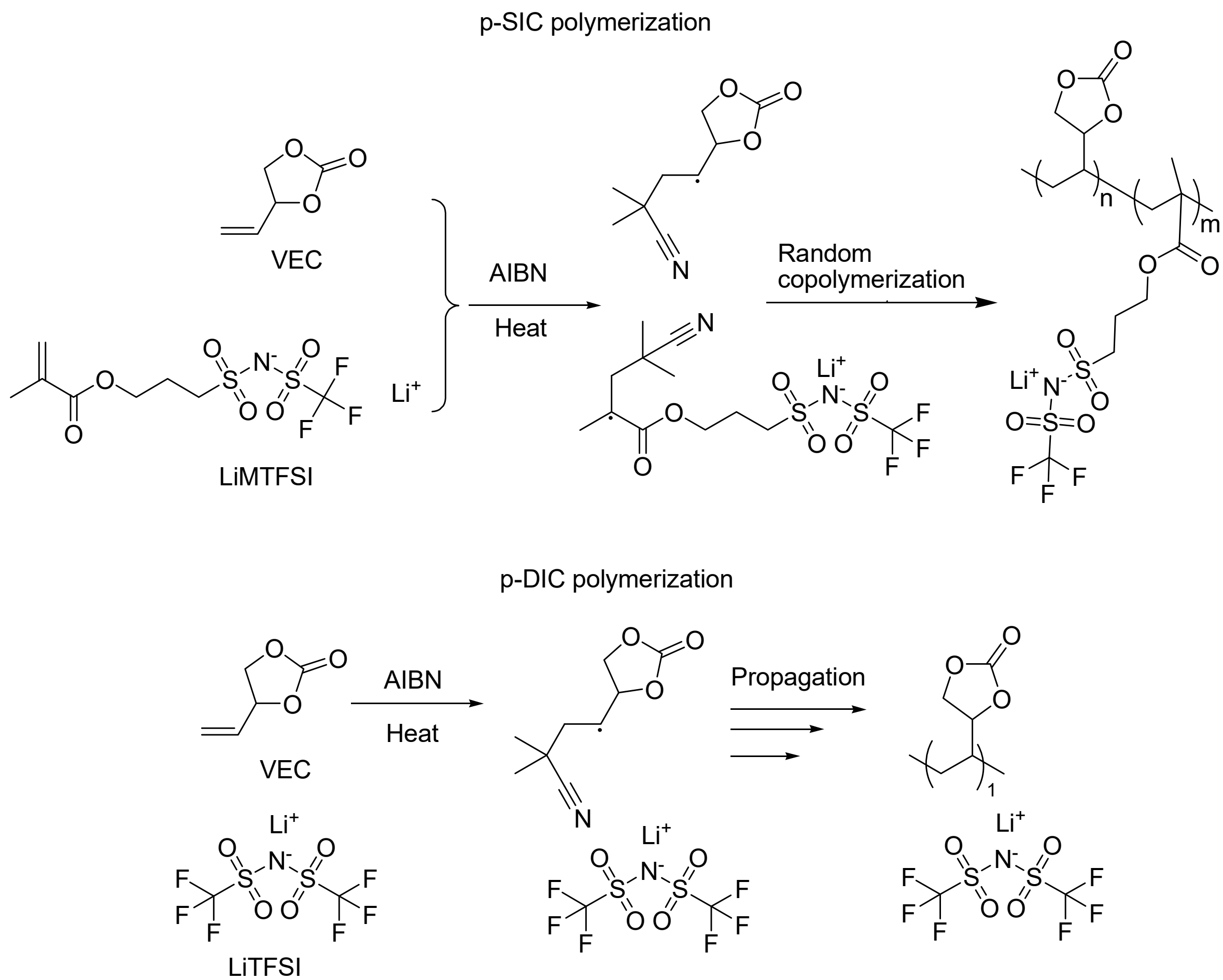

Figure S2. AIBN initiated polymerization reactions of Li single-ion-conductor (top, p-SIC) and dual-ion-conductor (bottom, p-DIC).

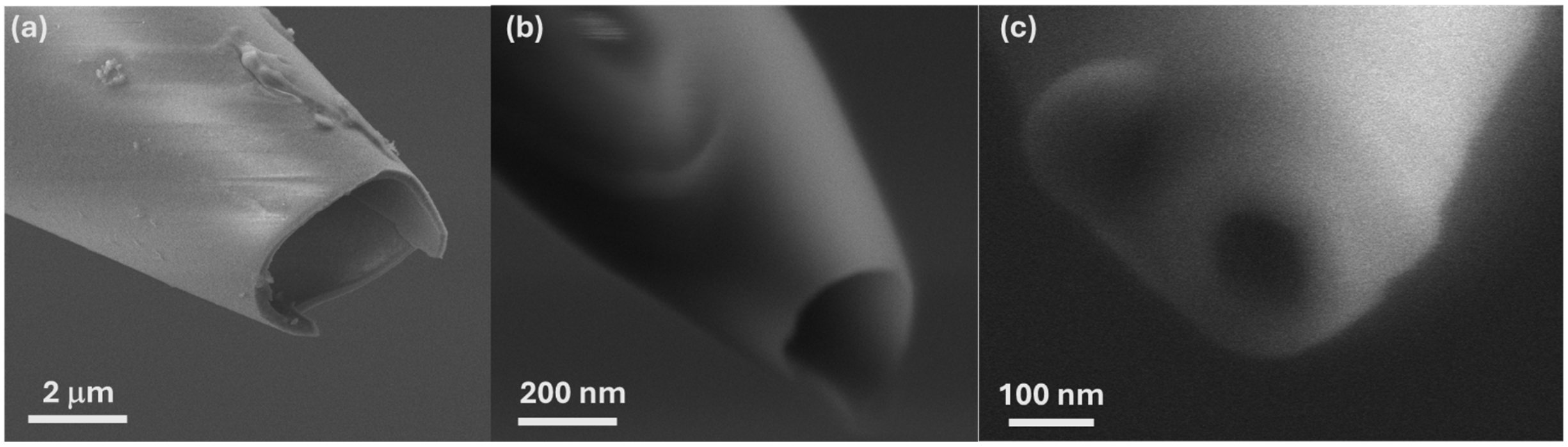

Figure S3. Additional SEM images of empty nano-micro-pipettes. SEM images of an empty micropipette (a) with R_i ~1.5 µm and nanopipettes (b) with R_i ~100 nm and (c) with R_i ~50 nm for comparison.

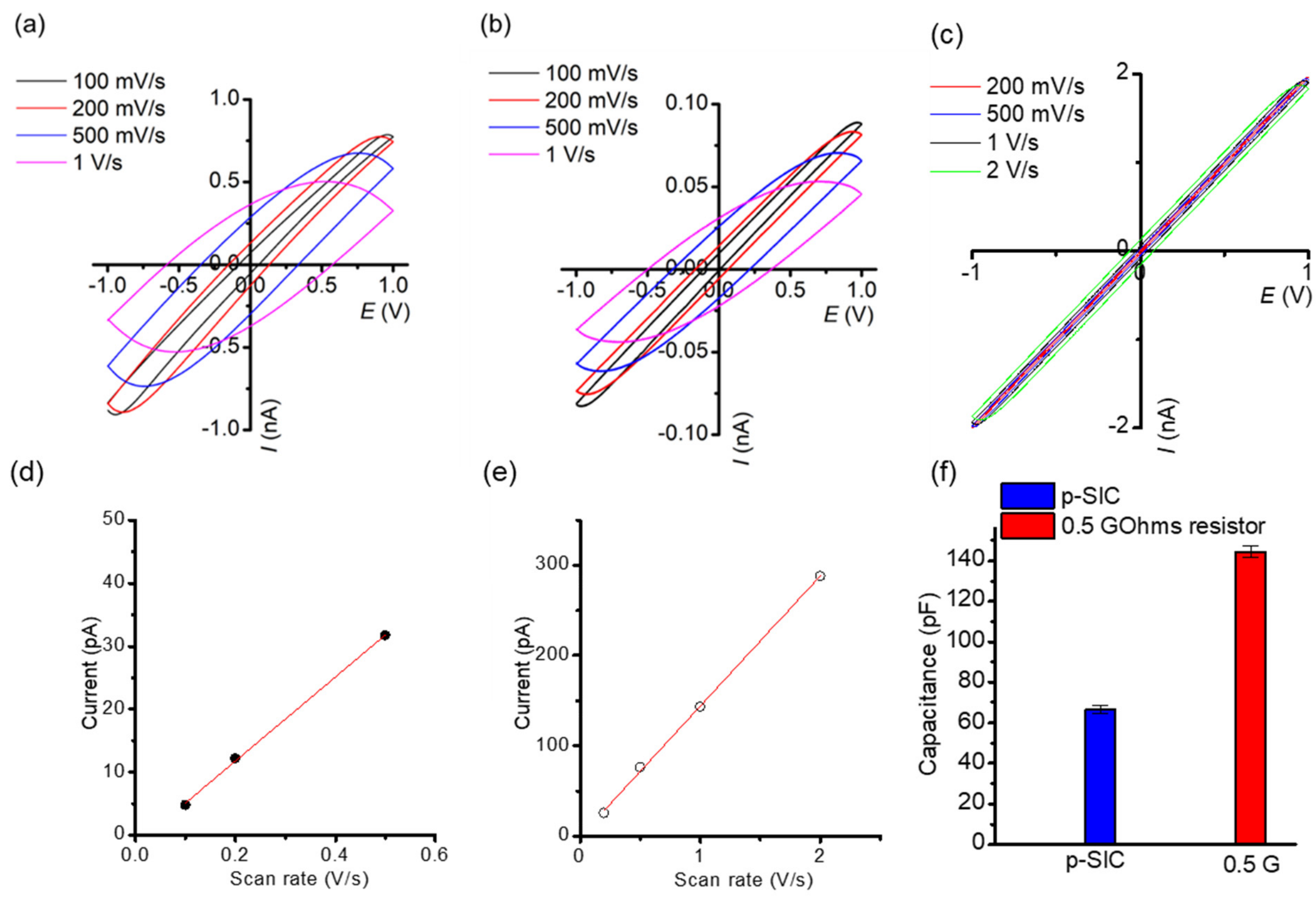

Figure S4. Additional CV results at different scan rates associated with SIC@NP polymerization. (a) *I-V* curves of pre-polymerized 10 mol% LiMTFSI/VEC in an NP (R_i ~100 nm) at scan rates from 100 mV/s to 1 V/s. (b) *I-V* curves of p-SIC filled NP (R_i ~100 nm) at scan rates from 100 mV/s to 1 V/s. (c) *I-V* curves of a 0.5 GΩ solid-state resistor at scan rates from 200 mV/s to 2 V/s for the evaluation of instrument and measurement parameters. Loop current as a function of scan rate from p-SIC filled NP (d) and 0.5 GΩ resistor (e). (f) Apparent capacitance of p-SIC filled NP (d) and stray capacitance when measuring a 0.5 GΩ resistor. The temperature of the measurements was room temperature, 25 °C.

The results suggest that the measured capacitance is in the same orders of magnitude with the stay capacitance of the measurement setup (although the largest solid-state resistor we have is still much smaller than the sample). Therefore, EIS analysis in Figure S7 is needed to evaluate the capacitive contributions to the DC conductivity.

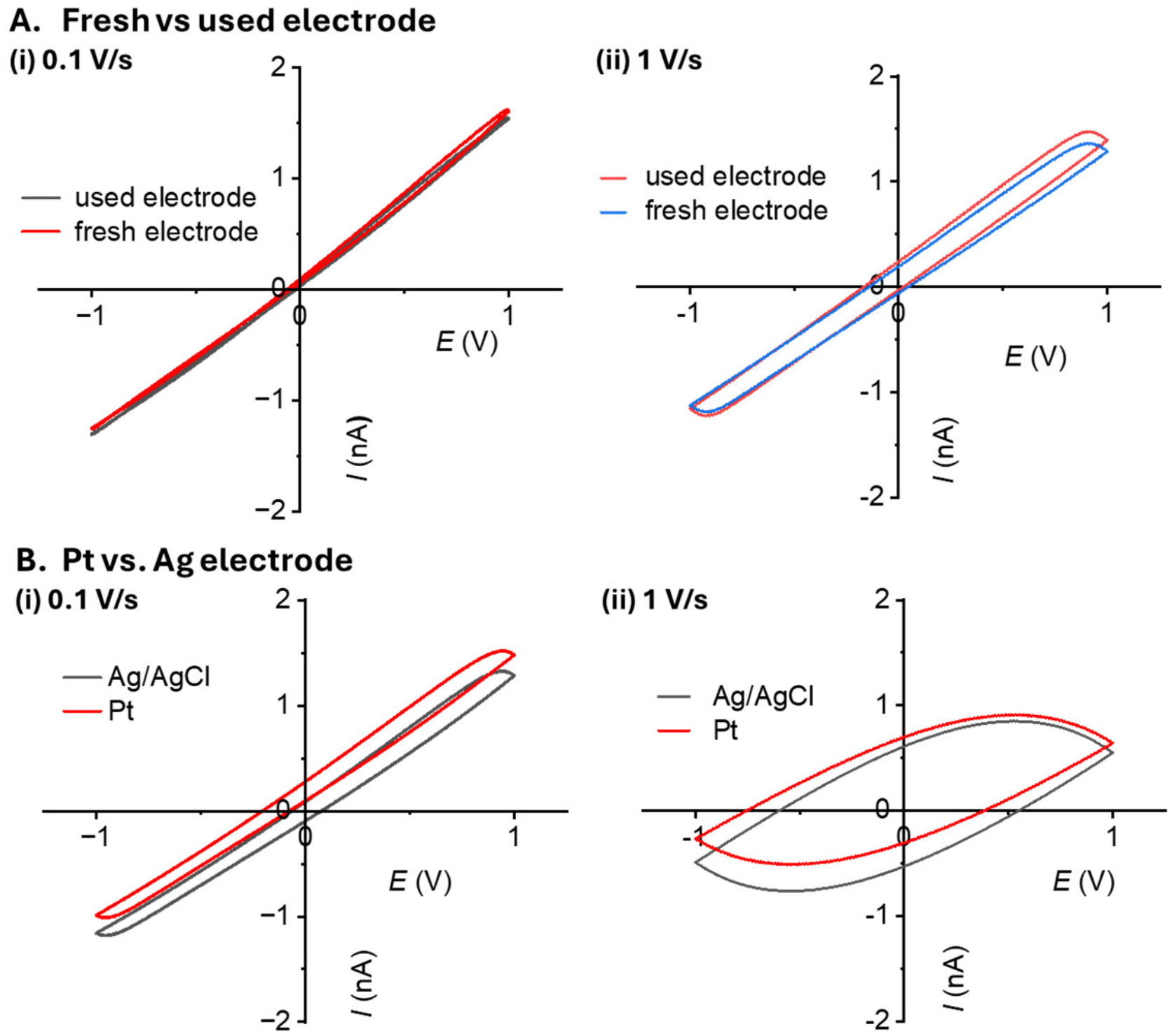

Figure S5. *I-V* curve comparison for (A) fresh vs used electrode and (B) Pt vs Ag/AgCl electrodes with two scan rates (i) 0.1 V/s and (ii) 1 V/s. Data obtained from a p-DIC NP at 23 °C in N_2 environment.

No significant difference in the *I–V* response was resolved between freshly prepared Ag/AgCl electrode and one used continuously for ~8 h. Additionally, negligible changes in current are observed when using different electrode materials (Pt vs Ag wires), indicating that the measurements are not sensitive to whether polarizable or nonpolarizable electrodes are used.

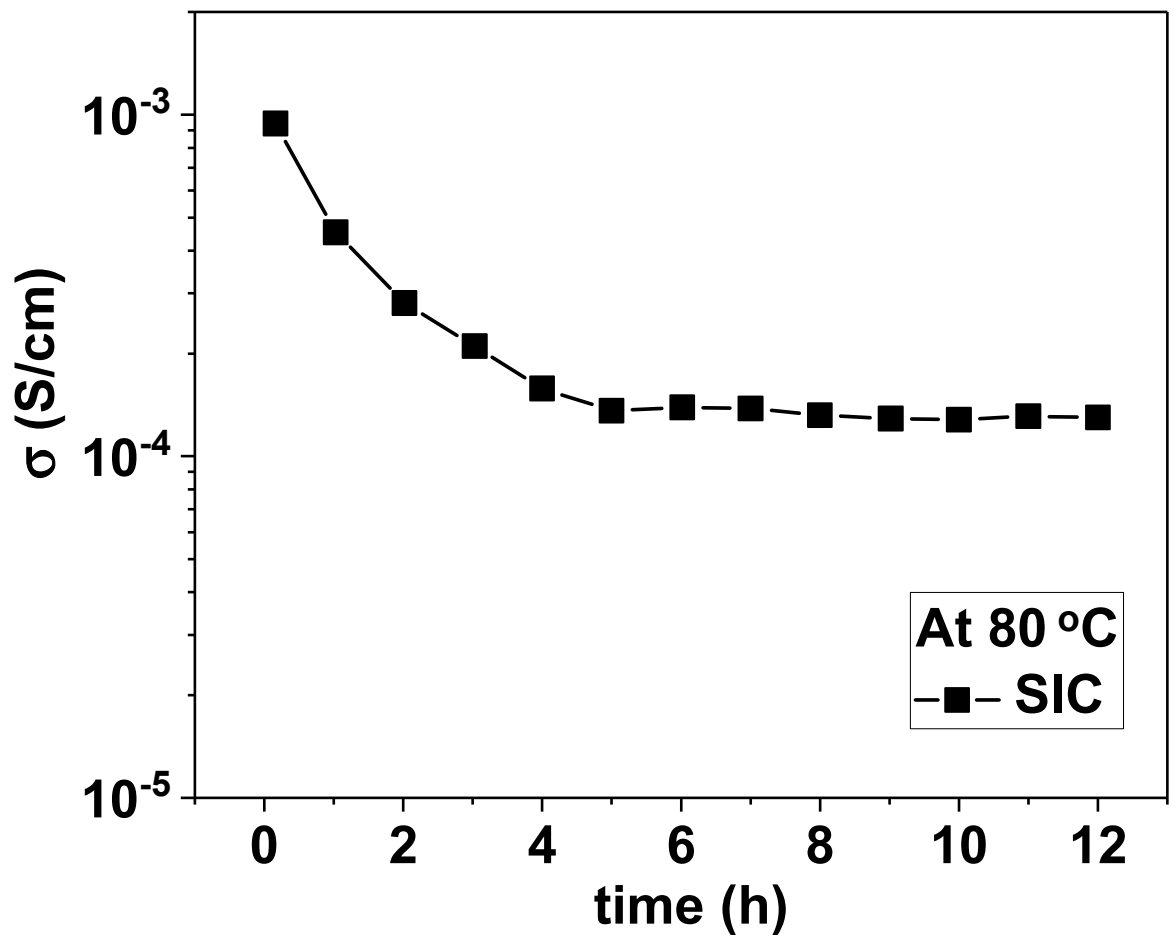

Figure S6. The evolution of ionic conductivity during SIC polymerization at 80 ºC from BDS analysis. Data recorded with an Alpha-A spectrometer. The samples were loaded into a spacer-free dielectric cell between two parallel electrode disks (diameter: 10.2 mm, gap: 0.3 mm). Each measurement was recorded after the system reached thermal equilibrium within 0.2 K.

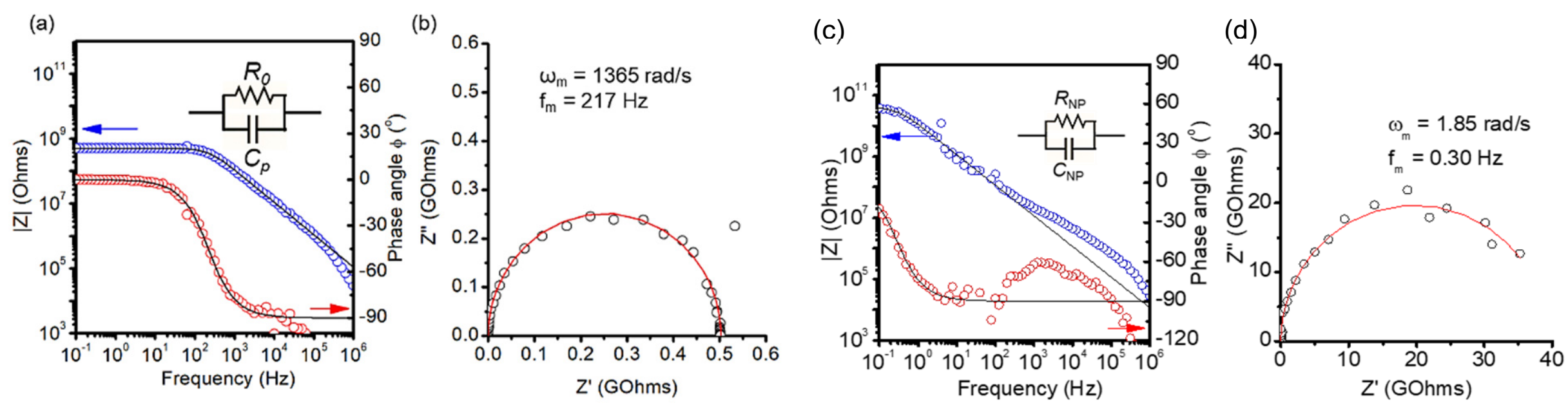

Figure S7. Electrochemical impedance spectroscopy (EIS) analysis of (a, b) a 0.5 GΩ (R_0) and (c, d) a p-SIC solid-solid (S/S) interface under the same measurement conditions. (a, c) are Bode plot and (b, d) are Nyquist plot. V_{dc} = 0 V and V_{ac} = 10 mV.

As this report focuses on the DC conductivity, a single R||C equivalent circuit was used to fit data in low frequency range. In reference to the parasitic stray capacitance C_p at about 1.4 pF using the solid-state resistor, the fittings of p-SIC@NP low frequency data result in the R_{NP} of 40 GΩ and C_{NP} of 14 pF. The high impedance of the sample is approaching the limits of the instrument and makes data in high frequency range unreliable for quantitative fitting, although adding another parallel *R*||*C* can drastically improve the fitting quality (not shown). Scattered data points in p-SIC@NP data in 10-100 Hz range are due to ground line frequency interference, i.e., inadequate Faraday shielding. The angular frequency and frequency associated with maximal *Z*'', ω_m and f_m, were labelled in the Nyquist plots.

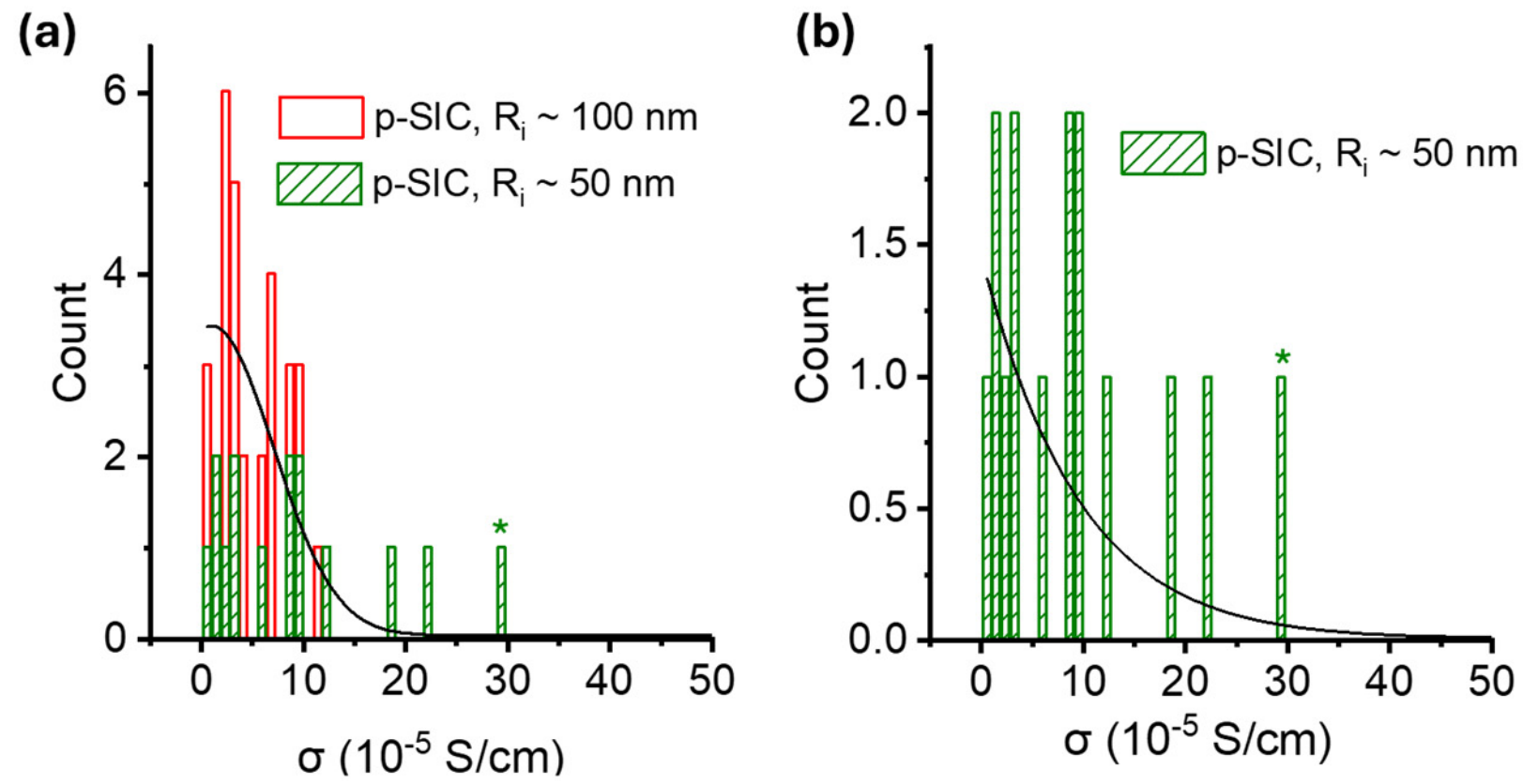

Figure S8. Gaussian fitting of conductivity of p-SIC NPs with (a) both R_i ~100 nm (#20) and R_i ~50 nm (#15) combined and (b) R_i ~50 nm only. Asterisk indicates outlier determined by G test.

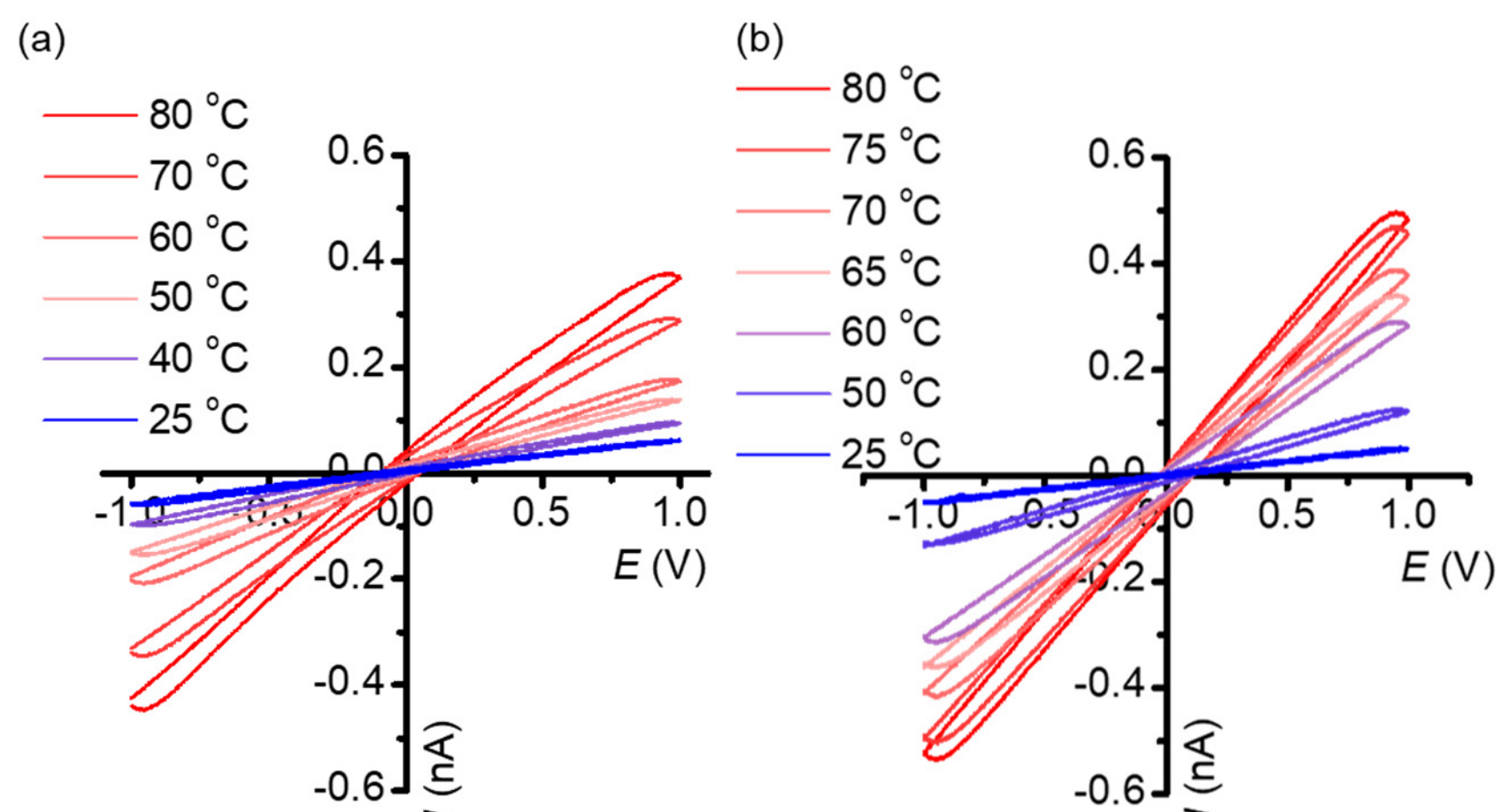

Figure S9. *I-V* curves of a p-SIC filled NP (R_i ~100 nm) at various temperatures. Panels (a) and (b) exhibit the thermal activation of S/S and S/L interfaces, respectively. Scan rate: 100 mV/s.

Table S2. Grubbs' test result of 35 p-SIC NPs with R_i~50 nm (# 1 to 15) and R_i~100 nm (# 16 to 35).

sample #	σ_{p-SIC} (S/cm)	G- Value	sample #	σ_{p-SIC} (S/cm)	G- Value
1	5.28E-06	0.97	18	2.00E-05	0.74
2	1.57E-05	0.81	19	2.00E-05	0.74
3	1.83E-05	0.76	20	2.10E-05	0.72
4	2.42E-05	0.67	21	2.50E-05	0.66
5	3.16E-05	0.55	22	2.50E-05	0.66
6	3.34E-05	0.52	23	2.80E-05	0.61
7	5.90E-05	0.11	24	3.10E-05	0.56
8	9.05E-05	0.4	25	3.50E-05	0.49
9	9.10E-05	0.41	26	4.10E-05	0.4
10	9.37E-05	0.45	27	4.30E-05	0.36
11	9.46E-05	0.47	28	5.60E-05	0.15
12	1.20E-04	0.88	29	6.70E-05	0.02
13	1.85E-04	1.93	30	6.70E-05	0.02
14	2.20E-04	2.49	31	7.00E-05	0.07
15	2.90E-04	3.62 *	32	7.10E-05	0.09
16	7.60E-06	0.94	33	8.20E-05	0.26
17	9.80E-06	0.9	34	9.40E-05	0.46
	** Outlier*		35	1.10E-05	0.72

15th *NP fall into outlier* (90% confidence) *based on G-test reference table.*[3] *The G- Value was calculated as,* $G = \frac{|X-\bar{X}|}{SD}$ *Where* $\bar{X}$ *is mean* $\sigma_{p\text{-SIC}}$ *(6.56×10^{-5} S/cm), X is* $\sigma_{p\text{-SIC}}$ *and SD is standard deviation (6.*19×*10^{-5} S/cm).* Iterative G-test results in the identification of successive outliers (all from NPs with R_i ~50 nm) upon removal of the previously detected outlier(s), until the sample count reduced to 32 (exclusion of 13th to 15th sample).

Table S3. Grubbs' test result of 15 p-SIC NPs with R_i ~50 nm.

sample #	σ_{p-SIC} (S/cm)	G- Value	sample #	σ_{p-SIC}(S/cm)	G- Value
1	5.28E-06	1.04	9	9.10E-05	0.01
2	1.57E-05	0.91	10	9.37E-05	0.03
3	1.83E-05	0.88	11	9.46E-05	0.04
4	2.42E-05	0.81	12	1.20E-04	0.34
5	3.16E-05	0.72	13	1.85E-04	1.13
6	3.34E-05	0.70	14	2.20E-04	1.55
7	5.90E-05	0.39	15	2.90E-04	2.39 *
8	9.05E-05	0.01		** Outlier*	

#15th NP (*) fall into outlier (90% confidence) based on G-test reference table.[3] Ca. mean $\sigma_{p\text{-}DIC}$ *9.15×10^{-5} S/cm and standard deviation 8.31×10^{-5} S/cm. There is no outlier for samples # 1 to 14 confirmed by the next iteration G-test.*

Table Error! No text of specified style in document.**S4.** Grubbs' test result of 32 p-DIC NPs with R_i 50 nm.

**sample count**	σ_{p-DIC} **(S/cm)**	**G- Value**	**sample count**	σ_{p-DIC} **(S/cm)**	**G- Value**
1	3.84E-05	5.33E-01	17	6.36E-04	2.54E-01
2	5.17E-05	5.27E-01	18	6.69E-04	2.39E-01
3	7.81E-05	5.15E-01	19	7.33E-04	2.09E-01
4	2.63E-04	4.28E-01	20	7.60E-04	1.96E-01
5	2.81E-04	4.20E-01	21	7.71E-04	1.91E-01
6	3.33E-04	3.96E-01	22	7.72E-04	1.91E-01
7	3.91E-04	3.69E-01	23	7.89E-04	1.83E-01
8	4.27E-04	3.52E-01	24	7.96E-04	1.79E-01
9	4.40E-04	3.46E-01	25	8.49E-04	1.55E-01
10	4.79E-04	3.28E-01	26	9.45E-04	1.10E-01
11	4.93E-04	3.21E-01	27	1.07E-03	5.14E-02
12	5.04E-04	3.16E-01	28	1.18E-03	0.00E+00
13	5.57E-04	2.91E-01	29	1.68E-03	2.34E-01
14	5.64E-04	2.88E-01	30	2.60E-03	6.64E-01
15	6.01E-04	2.71E-01	31	2.99E-03	8.46E-01
16	6.32E-04	2.56E-01	32	3.09E-03	8.93E-01

No outlier (90% confidence) *was found based on G-test reference table.[3] The G- Value was calculated as,* $G = \frac{|X-\bar{X}|}{SD}$ *Where* $\bar{X}$ *is mean* $\sigma_{p\text{-}DIC}$ *(1.18×10^{-3} S/cm), X is* $\sigma_{p\text{-}DIC}$ *and SD is standard deviation (2.14×10^{-3} S/cm).*

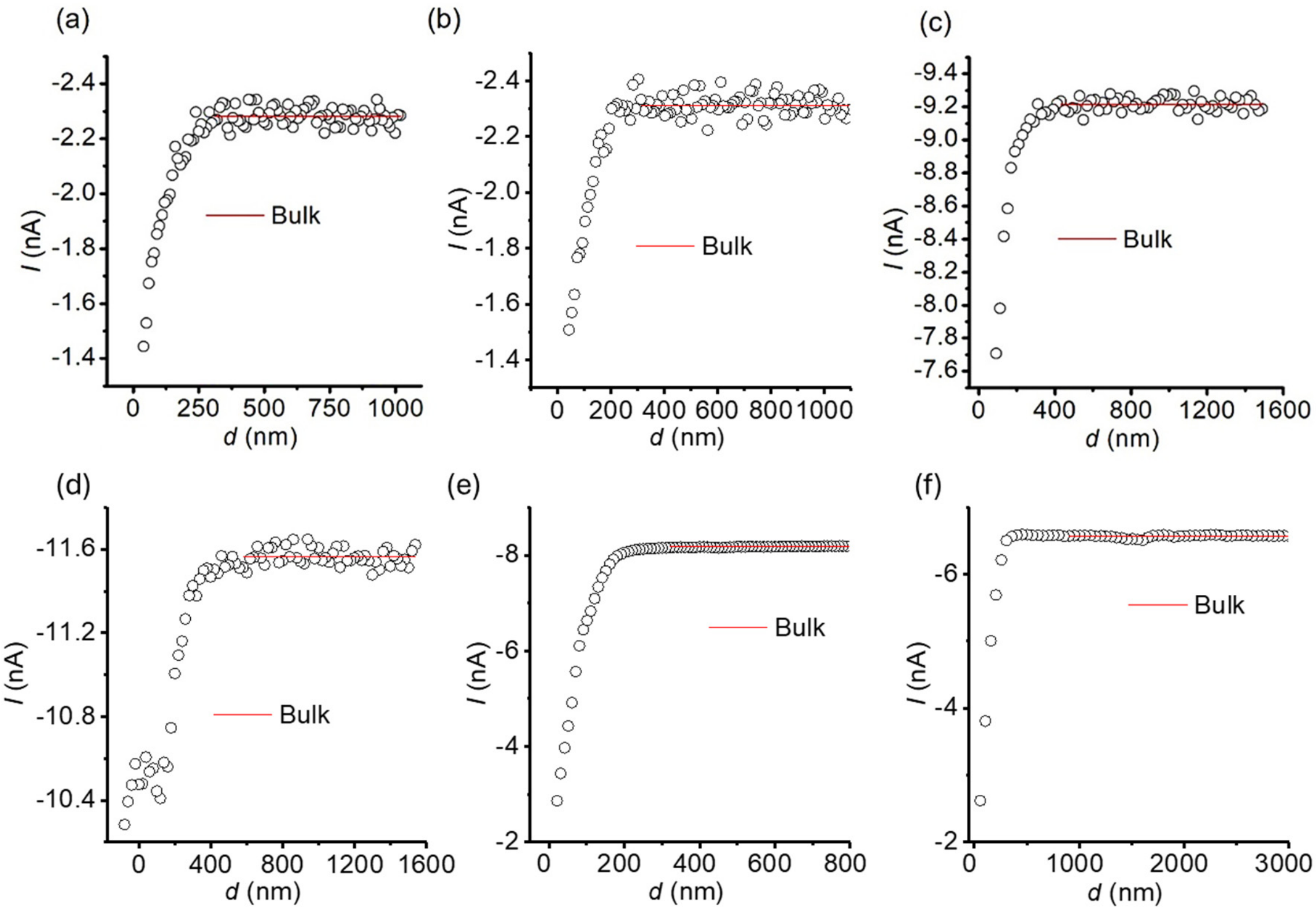

Figure S10. Approach curves (in original current) from additional polymerized p-DIC@NPs. Data from p-DIC NPs with different tip sizes (r_i) immersed in 10 mol% LiTFSI/VEC (1 M), plotted against tip-substrate distance (*d*). (a, b) 71 nm, (c) 283 nm, (d) 357 nm, (e) 252 nm and 202 nm were determined from the fitting of the bulk current (I_0). Piezo speed was 100 nm/s for (a) - (e) and 500 nm/s for (f) with step size of 10 nm for (a), (b) and (e), 20 nm for (c) and (d) and 50 nm for (f). Conditions: glass substrate, half-cone angle ~8° (SEM), σ_{p-DIC} = 7.5 × 10^{-4} S/cm, $\sigma_{LiTFSI/VEC}$ = 4.4 × 10^{-3} S/cm. The original current illustrates the noise level in each measurement and the steady-state current for the calculation of the tip radius. Note that data in (a) and Fig. 5C were from the same dataset. (a) and (b), (c) and (d), and (e) and (f) were two consecutive measurements from the same NP, respectively. Fitting statistics are in Table S5.

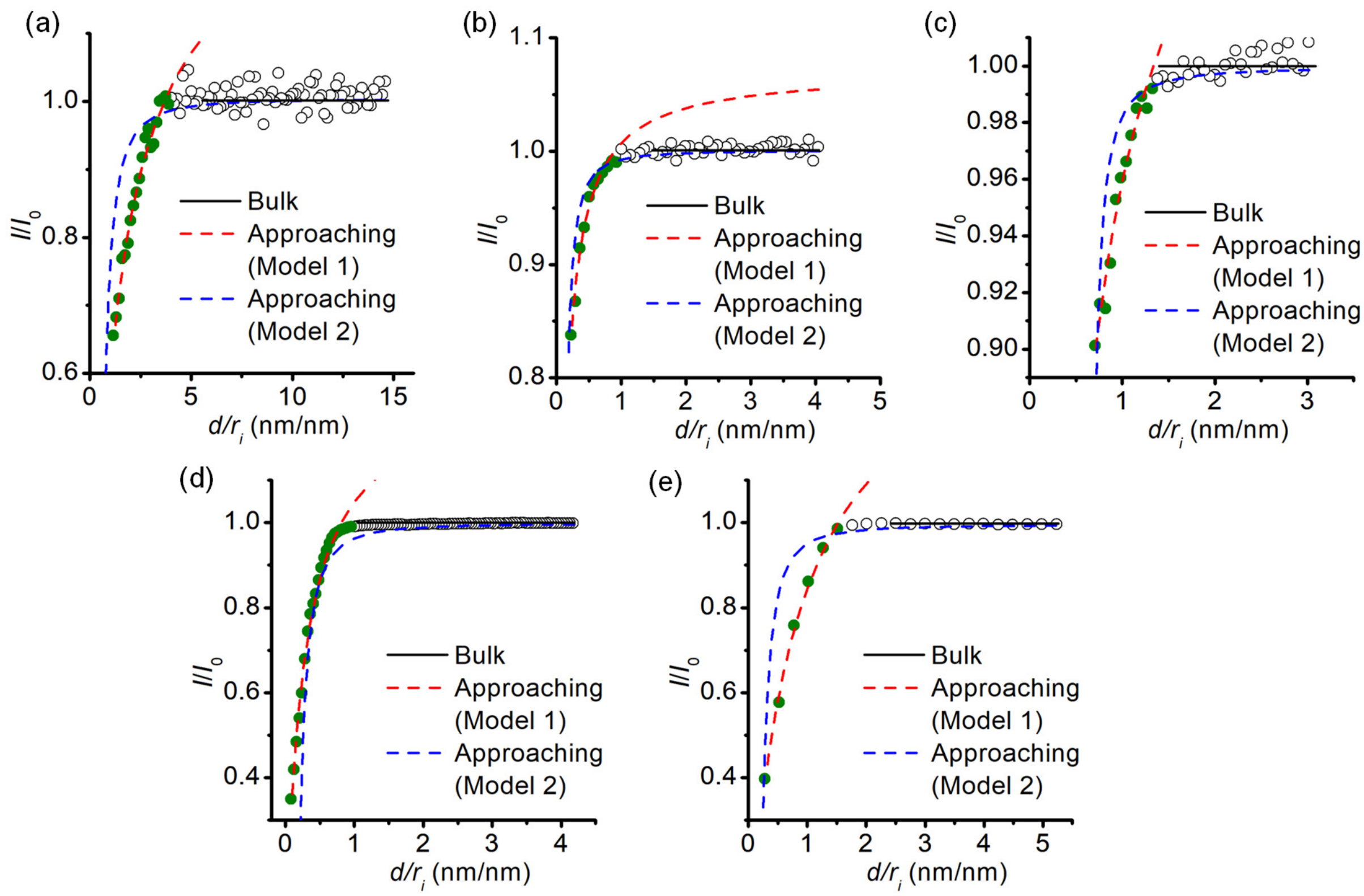

Figure S11. Approach curves in normalized tip current (I/I_0) using p-DIC@NPs. (a) 71 nm. (b) 283 nm. (c) 357 nm. (d) 252 nm. (e) 202 nm. Current below 99% of I_0 (solid circles, original data in Figure S10, panels b-f) was fitted using a model of distance-dependent ion current, I(d), provided in the method section. Note that data in (a) and Fig. 5C were from one NP; (b) and (c) from another NP and (d) and (e) were obtained from a third NP. Differences in I_0 or NP size are attributed to partial breakage (c) or tip contamination (e) which would affect the tip geometry and thus fitting. Fitting parameters: glass substrate, wall (D) of the NP ~25 nm, half-cone angle ~8° (SEM), $\sigma_{DIC} = 7.5 \times 10^{-4}$ S/cm, $\sigma_{LiTFS/VEC} = 4.4 \times 10^{-3}$ S/cm. Fitting results are summarized in Table S5.

Table S5. Summary of fitting parameters for p-DIC@NPs approach curves.

a. Radius calculation using **Eq. (3).**

**Fig. S10**	I_0 **(nA)**	r_i **(nm)**
a (data in Fig. 5c)	-2.28	70
b	-2.31	71
c	-9.21	283
d	-11.55	355
e	-8.19	252
f	-6.57	202

b. Fitting parameters using **Eq. (4)** without capacitance.

**Fig. S10**	Z_0 **(nm)**	**a**	**c**	R^2
*data in Fig. 5c	9029±9	0.13±0.02	0.90±0.02	0.98
a	9141±21	0.07±0.02	0.75±0.07	0.98
b	10962±20	0.30±0.10	0.93±0.02	0.99
c	11170±144	0.09±0.07	0.97±0.07	0.97
e	33261±3	0.10±0.01	0.75±0.02	0.99
f	256456±11	0.05±0.01	0.65±0.06	1.00

* (a)-(e) are from Fig. S10 except Fig. 5c.

c. Fitting parameters using **Eq. (5)** with capacitance.

**Fig. S10**	Z_0 **(nm)**	**b** $(nA \cdot nm^2)$	R^2
* data in Fig. 5c	9030±18	$2.7 \pm 1.1 \times 10^3$	0.71
a	9110±21	$2.7 \pm 1.4 \times 10^3$	0.64
b	10950±24	$4.4 \pm 2.6 \times 10^3$	0.86
c	10970±43	$3.4 \pm 4.0 \times 10^3$	0.42
e	33290±14	$1.7 \pm 0.6 \times 10^4$	0.87
f	256450±69	$1.1 \pm 2.0 \times 10^4$	0.61

* (a)-(e) are from Fig. S10 except Fig. 5c.

Table S6: Dependence of tip-substrate distance on fitting parameters*.

**Upper** I/I_0	**Lower** I/I_0	Z_0 **(nm)**	R^2
99	None	9030	0.71
99	70	9010	0.55
99	80	8980	0.49
95	None	9030	0.39
90	None	9030	0.3
85	None	9030	0.17

* Same dataset used in Fig. 5c and Table S5. The upper and lower ranges are varied in the fittings with **Eq. (5)**.

References:

(1) Brown, W.; Kvetny, M.; Yang, R.; Wang, G. Selective Ion Enrichment and Charge Storage through Transport Hysteresis in Conical Nanopipettes. *The Journal of Physical Chemistry C* **2022**, *126* (26), 10872-10879. DOI: 10.1021/acs.jpcc.2c02051.
(2) Brown, W.; Kvetny, M.; Yang, R.; Wang, G. Higher Ion Selectivity with Lower Energy Usage Promoted by Electro-osmotic Flow in the Transport through Conical Nanopores. *The Journal of Physical Chemistry C* **2021**, *125* (6), 3269-3276. DOI: 10.1021/acs.jpcc.0c08863.
(3) ASTM. *Standard Practice for Dealing with Outlying Observations*.
